# I_2_/DMSO-catalyzed one-pot approach for the synthesis of 1,3,4-selenadiazoles†

**DOI:** 10.1039/d0ra10576g

**Published:** 2021-02-02

**Authors:** Suresh Kuarm Bowroju, Rajitha Bavanthula

**Affiliations:** Department of Chemistry, National Institute of Technology Warangal TS India rajithabhargavi@ymail.com

## Abstract

A three-component cascade reaction for the synthesis of 1,3,4-selenadiazoles and their derivatives from arylaldehydes, hydrazine, and elemental selenium by using molecular iodine is reported. This strategy is operationally simple, well-suited to a wide range of functional groups, and provides the desired products in moderate to excellent yields. The proposed mechanism predicts that the reaction tolerated a radical process.

## Abstract 2

A three-component cascade reaction for the synthesis of 1,3,4-selenadiazoles and their derivatives from arylaldehydes, hydrazine, and elemental selenium by using molecular iodine is reported.

## Graphical abstract

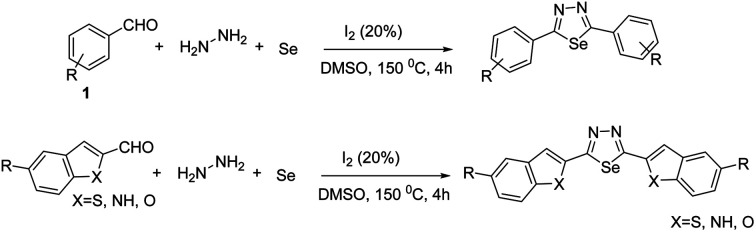

## Introduction

Selenium, is an element which plays a crucial role in biology and technology. It belongs to the chalcogen family, and is an essential element for living organisms as a component of selenomethionine, an amino acid occurring in the active centres of some enzymes, for example, glutathione peroxidase. Due to these significant properties, selenium-containing compounds such as biologically active molecules have been attractive to researchers. Organic and inorganic compounds of selenium also show unique electronic properties and are used as semi- and superconductors in electro-optic devices and sensors. The first organoselenium compound diethyl selenide was synthesized in 1836.^1^ 1,3,4-Selenadiazoles and their derivatives are closely related to the 1,3,4-thiadiazole compounds but their properties are quite different from the 1,3,4-thiadiazole compounds. 1,3,4-Selenadiazoles and their derivatives are applied widely in pharmaceutical, agricultural, and materials chemistry. 1,3,4-Selenadiazoles are important heterocycles with an “N–C–Se” linkage which can work as the active center, chelate certain metal ions *in vivo*, and show good tissue permeability. The lower toxicity and *in vivo* stability of the selenadiazole nucleus is attributed to its aromaticity. In particular, the 1,3,4-selenadiazoles have displayed a broad spectrum of biological activities including antibacterial, analgesic, antitumor, anticonvulsant, and anti-inflammatory drugs, pesticides and fungicides.^2–4^ Furthermore, some of them have been used as thermotropic liquid crystals, corrosion and oxidation inhibitors, or as dyes or metal ion complexation reagents.^5–10^ 1,3,4-Selenadiazole-containing kidney-type glutaminase inhibitors showed improved cellular uptake and antitumor activity.^11^ However, little is known about the 2,5-disubstituted 1,3,4-selenadiazoles.^12^

**Fig. 1:**
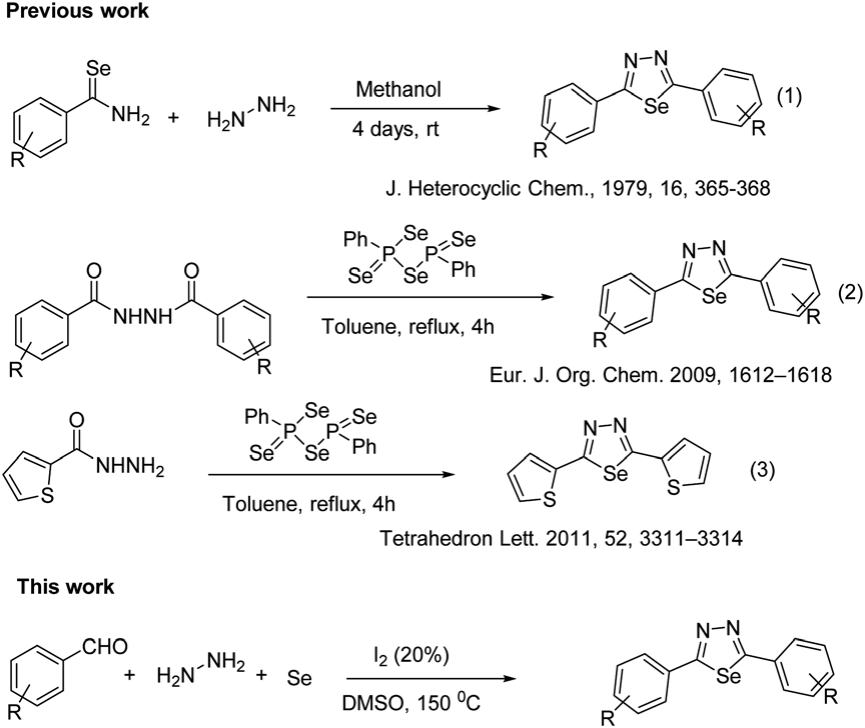
Methods for the synthesis of 1,3,4-selenadiazoles.

Several methods have been reported for their preparation, which include a ring-closure reaction of selenobenzamides with hydrazine hydrate,^13^ reacting dimethylformamide azine with hydrogen selenide,^14^ treatment of 1,2-diacetylhydrazine with phosphorus pentaselenide,^15^ reaction of isoselenocyanates with selenosemicarbazides^16^ or a carboxylic acid with selenosemicarbazide and phosphoryl chloride (Fig. 1).^17^ However, the examples of these compounds have some shortcomings in the literature are limited due to either lack of starting materials or very low yield. Therefore, developing an effective and low-cost method for synthesizing two-substituted 1,3,4-selenathiazoles is still highly warranted. In recent times, the iodine/DMSO combination has received considerable attention, in synthetic organic chemistry, as an effective and eco-friendly oxidative system as it has affected numerous organic transformations.^18–22^ As our continuous work on the development of a new method for the synthesis of various heterocyclic biologically active molecules,^23–43^ we herein report iodine/DMSO catalysed a three-component reaction for the preparation of 1,3,4-selenathiazoles from the easily available, aryl aldehyde, hydrazine and selenium powder.

## Results and discussion

Initially, our studies commenced using benzaldehyde 1a, hydrazine 2 and elemental selenium as a model substrate and the results are presented in Table 1. Firstly, 1a, 2 and selenium were taken in DMSO and the mixture was heated at 150 °C for 4 hours, however, the reaction failed to proceed and no product was detected (Table 1, entry 1). Due to the failure of this reaction, confirming the importance of catalyst in this multicomponent reaction. Therefore, we focused on sequential additions and accordingly, we attempted a reaction with 1a, 2, selenium and TBAI in DMSO, and heated at 150 °C for 4 hours. Encouragingly, the expected product 3a was obtained in an isolated yield of 51% (Table 1, entry 2). Accordingly, we focused our studies on to improve the yield, the reaction was attempted with KI and NH_4_I affording 3a in 42% and 35% yield respectively (Table 1, entries 3 and 4). Finally, I_2_ was shown to be the best performing catalyst as the yield significantly increased to 88% (Table 1, entry 7). Many synthetic procedures use varying amounts of I_2_ and reaction temperatures depending on the reaction conditions employed to produce the desired product in maximum yield. Thus, to complete the study, attempts were made to decrease the amount of iodine to 5% and 10%, however, lowered the yield of the desired product to 71% and 82% respectively (Table 1, entries 5 and 6). Furthermore, attempt was made to increase the amount of iodine to 30%, however, lowered the yield of the desired product to 79% (Table 1, entry 8). Further, the attempt was made to decrease the temperature to 120 °C, however, lowered the yield of the desired product to 76% (Table 1, entry 9). Finally, the attempt was made to increase the temperature to 170 °C, however, lowered the yield of the desired product to 76% (Table 1, entry 10). To conclude, the conditions described in entry 7 were found to be optimal, allowing for maximum conversion to the desired product 3a.

**Table 1:** Optimization of the reaction conditions^a^

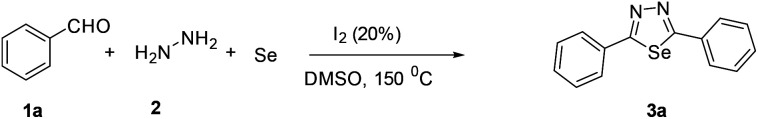				
Entry	Catalyst (%)	Solvent	Temp. (0 °C)	Yield (%)
1	—	DMSO	150	Trace
2	TBAI (20)	DMSO	150	51
3	NH_4_I (20)	DMSO	150	35
4	KI (20)	DMSO	150	42
5	I_2_ (5)	DMSO	150	71
6	I_2_ (10)	DMSO	150	82
7	I_2_ (20)	DMSO	150	88
8	I_2_ (30)	DMSO	150	79
9	I_2_ (20)	DMSO	120	76
10	I_2_ (20)	DMSO	170	71

a 1a (0.5 mmol), 2 (0.25 mmol), Se (0.25 mmol), catalyst (20 mol%), and solvent (3 mL) under air at 150 °C for 4 h.

**Scheme 1:**
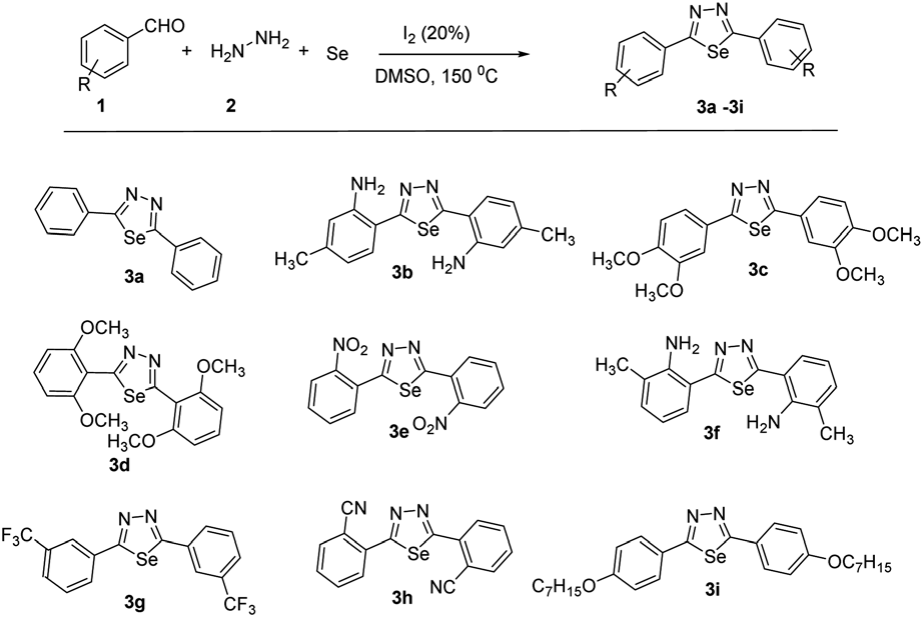
Synthesis of 2,5-diaryl-1,3,4-selenadiazoles.

**Scheme 2:**
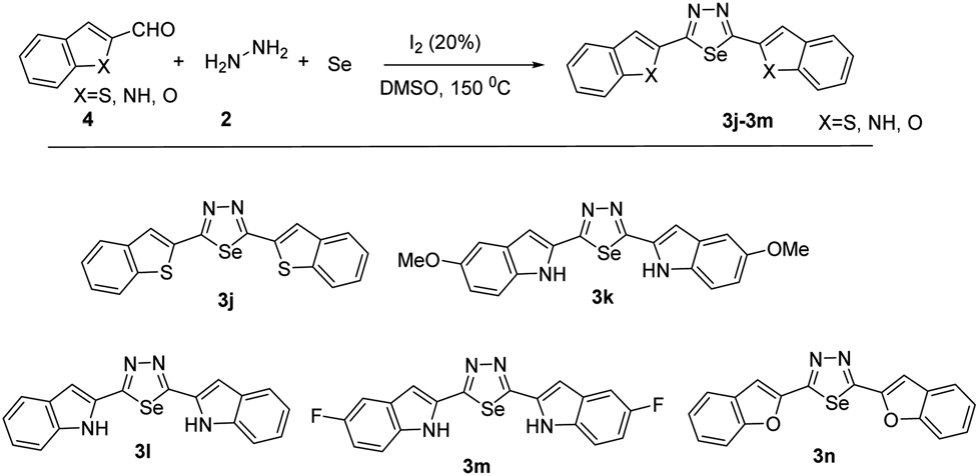
Synthesis of 2,5-diheteroaryl-1,3,4-selenadiazoles.

With the optimized reaction conditions in hand (Table 1, entry 7), we subsequently investigated the substrate scope of this transformation (Schemes 1 and 2). Benzaldehyde bearing neutral, electron-donating, and electron-withdrawing substituents at the *o*/*m*/*p*-positions of the aromatic ring reacted smoothly under the optimized reaction conditions, affording the corresponding product in high yields (73–92%). Besides different benzaldehydes, other benzothiophene, benzofuran, indole aldehydes also yields 65–74%.

Moreover, the reaction also tolerated several functional groups, such as nitrile, methoxy, iodo and amino. All reactions proceeded smoothly to give the desired products in moderate to good yields. The reactivities of phenyl rings with strongly electron-withdrawing substituents (–CF_3_, –NO_2_) were higher than those of phenyl rings with electron-donating substituents. This suggests that electron-withdrawing groups enhance the reaction efficiency (3e and 3g). Substrates with substituents at the *ortho* or *meta* positions were also compatible with. Notably, the position of the substituent did not affect the reaction productivities and yields (3c, 3d and 3b, 3f). After replacing the phenyl group with heteroaryl, such as benzothiophene, benzofuran, indole aldehydes. All reactions proceeded smoothly to give the desired products in moderate to good yields. Compared with indole aldehydes, benzothiophene, benzofuran showed a lower reactivity in the reaction.

**Scheme 3:**
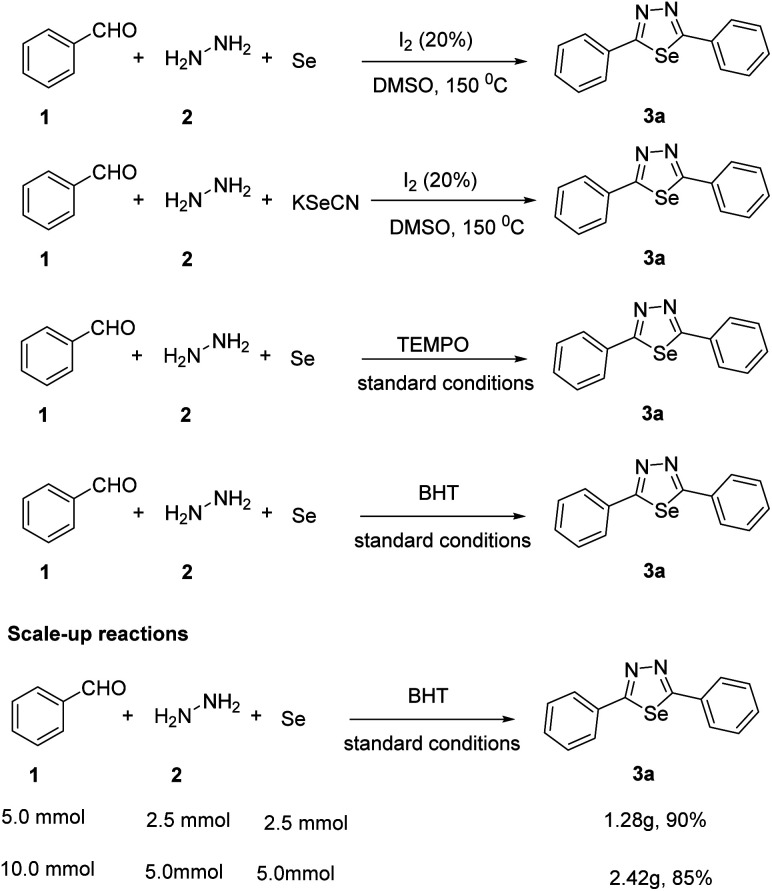
Controlled experiments.

We have performed the several control experiments to interpret the reaction mechanism (Scheme 3). The first control experiment was carried out with selenium powder and synthetic diimine, it could be converted into the corresponding desired product 3a in 92% yield. This reaction completed in 1 h. This is because the process is one-step less than the standard reaction. Later, when we replaced Se with KSeCN, desired product 3a was obtained in moderate yield. Finally, radical inhibitors tetramethyl-piperidin-1-oxyl (TEMPO) and butylated hydroxytoluene (BHT) were used resulted in the inhibition of the reaction. These results suggested that this reaction proceeded through a radical pathway. We performed the scale up reactions 5 mmol and 10 mmol and obtained the desired product in 90% and 85% yields, respectively.

**Scheme 4:**
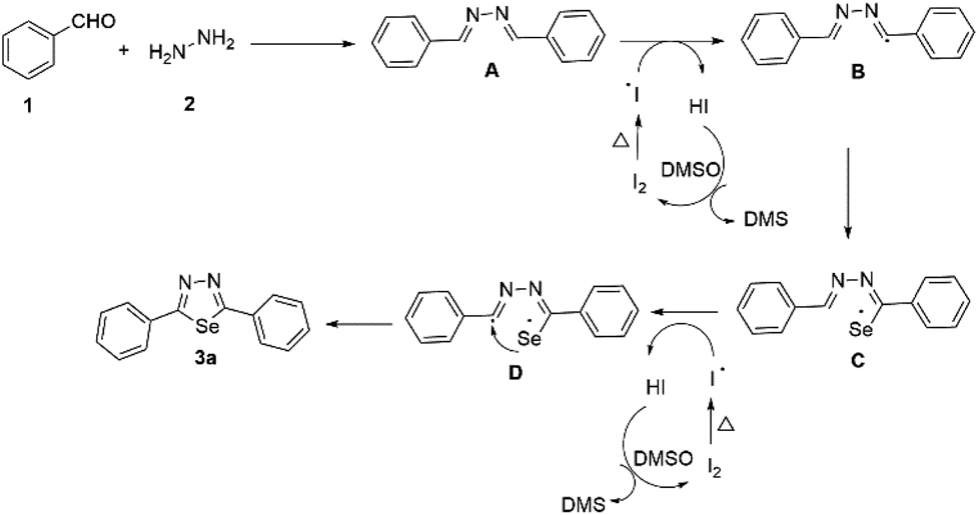
Proposed mechanism.

From the above results, a proposed mechanism is presented in Scheme 4. Initially, di-imine generated from benzaldehyde 1a and hydrazine 2. Iodine radicals produced upon heating the molecular iodine. Then, iodine radicals get a proton from A to obtain the radical intermediate B and HI. HI reacts with DMSO to generate iodine, and the cycle continues.^44,45^ Later, radical intermediate B reacts with selenium powder to afford the selenium free-radical intermediate C.^46^ Again iodine radicals get a proton from C to obtain the radical intermediate D and HI. Finally, intermediate D undergoes cyclization to furnish the desired product 3a.

## Conclusion

We have successfully developed a practical way for the synthesis of 1,3,4-selenadiazole through I_2_/DMSO promoted cascade cyclization process. We reported a mild method by the multicomponent reaction of different aldehydes, elemental selenium, and hydrazine. The present methodology has the advantages of being free of metal, the use of simple operation.

## Experimental section

### General procedure for the synthesis of 2,5-diaryl-1,3,4-selenadiazoles

Aldehyde (0.5 mmol), elemental selenium (0.25 mmol), hydrazine (0.25 mmol), iodine (20%), and DMSO (3 mL) were added to the reaction vial. The mixture was stirred at 150 °C for 4 h. After completion of reaction (confirmed by TLC), water was added and extracted with ethyl acetate. The combined organic layers were dried over magnesium sulfate. Evaporation of the solvent under reduced pressure provided the crude product, which was purified by column chromatography on silica gel (1–3% MeOH in dichloromethane as eluent) to give the corresponding target product.

### 2,5-Diphenyl-1,3,4-selenadiazole (3a)

White solid; Yield = 88%; mp 115–117 °C (lit. mp 116–118 °C);^47^^1^H̲ NMR (400 MHz, DMSO-d_6_): *δ* 7.85 (d, *J* = 7.8 Hz, 4H), 7.21–7.22 (m, 6H); ^13^C̲ NMR (400 MHz, DMSO-d_6_): *δ* 170.75, 140.41, 129.51, 129.44, 129.14, 127.47, 109.55; HRMS (ESI) *m*/*z* calcd for C_14_H_10_N_2_Se (M + H)^+^ 287.0269, found 287.0300; elemental analysis: C, 58.96; H, 3.53; N, 9.82; Se, 27.69, found: C, 58.86; H, 3.62; N, 9.87; Se, 27.65.

### 6,6′-(1,3,4-Selenadiazole-2,5-diyl)bis(3-methylaniline) (3b)

Brown solid; yield = 73%; mp 165–167 °C; ^1^H̲ NMR (400 MHz, CDCl_3_): *δ* 7.79 (d, *J* = 8.0 Hz, 2H), 6.47 (s, 2H), 6.44 (brs, 2H), 2.25 (s, 6H); ^13^C̲ NMR (400 MHz, CDCl_3_): 173.58, 151.03, 145.91, 132.03, 118.04, 116.86, 107.49, 21.67; HRMS (ESI) *m*/*z* calcd for C_16_H_16_N_4_Se (M + H)^+^ 345.0581, found 345.0565; elemental analysis: C, 55.98; H, 4.70; N, 16.32; Se, 23.00, found C, 55.91; H, 4.72; N, 16.4; Se, 22.97.

### 2,5-Bis(3,4-dimethoxyphenyl)-1,3,4-selenadiazole (3c)

White solid; yield = 84%; mp 210–212 °C; ^1^H̲ NMR (400 MHz, CDCl_3_): *δ* 7.74 (d, *J* = 8.0 Hz, 2H), 7.74 (s, 2H), 6.9 (d, *J* = 8.0 Hz, 2H), 3.93 (s, 6H), 3.92 (s, 6H); ^13^C̲ NMR (400 MHz, CDCl_3_): 172.04, 153.71, 148.64, 124.58, 121.67, 112.26, 110.28, 56.05, 55.95; HRMS (ESI) *m*/*z* calcd for C_18_H_18_N_2_O_4_Se (M + H)^+^ 407.0155, found 407.0141; elemental analysis: C, 53.34; H, 4.48; N, 6.91; O, 15.79; Se, 19.48, found C, 53.31; H, 4.50; N, 6.92; O, 15.68; Se, 19.59.

### 2,5-Bis(2,6-dimethoxyphenyl)-1,3,4-selenadiazole (3d)

White solid; yield = 85%; mp 223–225 °C; ^1^H̲ NMR (400 MHz, CDCl_3_): *δ* 7.33 (d, *J* = 8.0 Hz, 2H), 6.55 (d, *J* = 8.0 Hz, 4H), 3.85 (s, 12H); ^13^C̲ NMR (400 MHz, CDCl_3_): 170.69, 157.81, 131.82, 111.59, 104.1, 56.16, 56.14; HRMS (ESI) *m*/*z* calcd for C_18_H_18_N_2_O_4_Se (M + H)^+^ 407.0155, found 407.0141; elemental analysis: C, 53.34; H, 4.48; N, 6.91; O, 15.79; Se, 19.48, found C, 53.29; H, 4.51; N, 6.89; O, 15.81; Se, 19.50.

### 2,5-Bis(2-nitrophenyl)-1,3,4-selenadiazole (3e)

Pale yellow solid; yield = 90%; mp 265–267 °C; ^1^H̲ NMR (400 MHz, CDCl_3_): *δ* 8.06–8.00 (m, 4H), 7.45 (t, *J* = 8.0 Hz, 2H), 7.21 (d, *J* = 7.6 Hz, 2H); ^13^C̲ NMR (400 MHz, CDCl_3_): 171.31, 141.98, 133.57, 133.09, 132.1, 128.02, 94.71; HRMS (ESI) *m*/*z* calcd for C_14_H_8_N_4_O_4_Se (M + H)^+^ 376.9842, found 376.9870; elemental analysis: C, 44.82; H, 2.15; N, 14.93; O, 17.06; Se, 21.04, found C, 44.75; H, 2.18; N, 14.89; O, 17.10; Se, 21.08.

### 6,6′-(1,3,4-Selenadiazole-2,5-diyl)bis(2-methylaniline) (3f)

Light brown solid; yield = 78%; mp 188–190 °C; ^1^H̲ NMR (400 MHz, DMSO-d_6_): *δ* 7.63 (d, *J* = 8.0 Hz, 2H), 7.16 (d, *J* = 7.2 Hz, 2H), 6.48 (t, *J* = 8.0 Hz, 2H), 2.09 (s, 6H); ^13^C̲ NMR (400 MHz, DMSO-d_6_): 172.69, 152.34, 137.04, 131.66, 125.6, 116.94, 112.01, 20.13; HRMS (ESI) *m*/*z* calcd for C_16_H_16_N_4_Se (M + H)^+^ 345.0481, found 345.0490; elemental analysis: C, 55.98; H, 4.70; N, 16.32; Se, 23.00, found C, 55.92; H, 4.71; N, 16.4; Se, 22.97.

### 2,5-Bis(3-(trifluoromethyl)phenyl)-1,3,4-selenadiazole (3g)

White solid; yield = 92%; mp 155–157 °C; ^1^H̲ NMR (400 MHz, DMSO-d_6_): *δ* 8.23 (s, 2H), 8.17 (d, *J* = 7.6 Hz, 2H), 8.03 (d, *J* = 7.6 Hz, 2H), 7.67 (t, *J* = 8.0 Hz, 2H); ^13^C̲ NMR (400 MHz, DMSO-d_6_): 166.10, 136.62, 134.16, 133.17, 132.48, 130.43, 118.48, 112.34; HRMS (ESI) *m*/*z* calcd for C_16_H_8_F_6_N_2_Se (M + H)^+^ 422.9569, found 422.9550; elemental analysis: C, 45.62; H, 1.91; F, 27.06; N, 6.65; Se, 18.75, found C, 45.58; H, 1.88; F, 27.04; N, 6.71; Se, 18.79.

### 2,2′-(1,3,4-Selenadiazole-2,5-diyl)dibenzonitrile (3h)

Off-white solid; yield = 80%; mp 159–160 °C; ^1^H̲ NMR (400 MHz, DMSO-d_6_): *δ* 8.05 (d, *J* = 8.0 Hz, 2H), 8.02 (d, *J* = 6.8 Hz, 2H), 7.76–7.72 (m, 4H); ^13^C̲ NMR (400 MHz, DMSO-d_6_): 165.68, 134.42, 133.76, 133.56, 133.32, 131.33, 118.16, 112.07; HRMS (ESI) *m*/*z* calcd for C_16_H_8_N_4_Se (M + H)^+^ 336.9953, found 336.9972; elemental analysis: C, 57.33; H, 2.41; N, 16.71; Se, 23.55, found: C, 57.30; H, 2.44; N, 16.76; Se, 23.50.

### 2,5-Bis(4-(heptyloxy)phenyl)-1,3,4-selenadiazole (3i)

White solid; yield = 81%; mp 95–98 °C; ^1^H̲ NMR (400 MHz, DMSO-d_6_): *δ* 7.84 (d, *J* = 8.8 Hz, 4H), 6.96 (d, *J* = 8.8 Hz, 4H), 3.99 (t, *J* = 6.4 Hz, 4H), 1.69–1.65 (m, 4H), 1.35–1.22 (m, 16H), 0.83 (t, *J* = 6.8 Hz, 6H); ^13^C̲ NMR (400 MHz, DMSO-d_6_): 167.43, 162.72, 131.78, 123.19, 114.62, 68.17, 31.65, 28.96, 28.83, 25.83, 22.47, 14.36; HRMS (ESI) *m*/*z* calcd for C_28_H_38_N_2_O_2_Se (M + H)^+^ 515.2109, found 515.2095; elemental analysis: C, 65.48; H, 7.46; N, 5.45; O, 6.23; Se, 15.37, found: C, 65.54; H, 7.44; N, 5.41; O, 6.19; Se, 15.42.

### 2,5-Di(benzofuran-2-yl)-1,3,4-selenadiazole (3j)

White solid; yield = 70%; mp 270–272 °C; ^1^H̲ NMR (400 MHz, DMSO-d_6_): *δ* 7.75 (d, *J* = 8.0 Hz, 2H), 7.66 (d, *J* = 8.0 Hz, 2H), 7.62 (s, 2H), 7.47 (t, *J* = 8.4 Hz, 2H), 7.32 (t, *J* = 8.0 Hz, 2H); ^13^C̲ NMR (400 MHz, DMSO-d_6_): 160.53, 155.4, 146.61, 127.98, 127.28, 124.24, 123.52, 113.95, 113.88, 112.49; HRMS (ESI) *m*/*z* calcd for C_18_H_10_N_2_O_2_Se (M + H)^+^ 366.9939, found 366.9916; elemental analysis: C, 59.19; H, 2.76; N, 7.67; O, 8.76; Se, 21.62, found: C, 59.15; H, 2.82; N, 7.65; O, 8.71; Se, 21.67.

### 2,5-Bis(benzo[*b*]thiophen-2-yl)-1,3,4-selenadiazole (3k)

White solid; yield = 65%; mp 265–268 °C; ^1^H̲ NMR (400 MHz, DMSO-d_6_): *δ* 8.07 (s, 2H), 8.0–7.95 (m, 4H), 7.46–7.41 (m, 4H); ^13^C̲ NMR (400 MHz, DMSO-d_6_): 163.96, 141.74, 139.15, 135.15, 130.7, 130.65, 127.44, 126.13, 125.49, 123.38; HRMS (ESI) *m*/*z* calcd for C_18_H_10_N_2_S_2_Se (M + H)^+^ 399.9454, found 399.9444; elemental analysis: C, 54.41; H, 2.54; N, 7.05; S, 16.14; Se, 19.87, found: C, 54.39; H, 2.51; N, 7.11; S, 16.10; Se, 19.89.

### 2,5-Di(1*H*-indol-2-yl)-1,3,4-selenadiazole (3l)

White solid; yield = 73%; mp 289–290 °C; ^1^H̲ NMR (400 MHz, DMSO-d_6_): *δ* 11.71 (s, 2H), 7.61 (d, *J* = 8.4 Hz, 2H), 7.41 (d, *J* = 8.4 Hz, 2H), 7.21 (t, *J* = 8.4 Hz, 2H), 7.06 (s, 2H), 7.05 (t, *J* = 8.0 Hz, 2H); ^13^C̲ NMR (400 MHz, DMSO-d_6_): *δ* 163.25, 137.66, 128.83, 127.28, 124.7, 122.34, 120.38, 112.91, 107.72; HRMS (ESI) *m*/*z* calcd for C_18_H_12_N_4_Se (M + H)^+^ 365.0279, found 365.0296; elemental analysis: C, 59.51; H, 3.33; N, 15.42; Se, 21.74, found: C, 59.47; H, 3.32; N, 15.39; Se, 21.82.

### 2,5-Bis(5-methoxy-1*H*-indol-2-yl)-1,3,4-selenadiazole (3m)

White solid; yield = 74%; mp 289–290 °C; ^1^H̲ NMR (400 MHz, DMSO-d_6_): *δ* 11.57 (s, 2H), 7.3 (d, *J* = 8.8 Hz, 2H), 7.05 (s, 2H), 6.96 (s, 2H), 6.87 (dd, *J* = 8.8 Hz, *J* = 2.4 Hz, 2H), 3.71 (s, 6H),; ^13^C̲ NMR (400 MHz, DMSO-d_6_): 161.17, 154.24, 132.99, 129.03, 127.57, 116.21, 113.78, 107.36, 102.38, 55.66; HRMS (ESI) *m*/*z* calcd for C_20_H_16_N_4_O_2_Se (M + H)^+^ 425.0442, found 425.0458; elemental analysis: C, 56.74; H, 3.81; N, 13.23; O, 7.56; Se, 18.65, found: C, 56.69; H, 3.82; N, 13.27; O, 7.63; Se, 18.59.

### 2,5-Bis(5-fluoro-1*H*-indol-2-yl)-1,3,4-selenadiazole (3n)

White solid; yield = 71%; mp 268–270 °C; ^1^H̲ NMR (400 MHz, DMSO-d_6_): *δ* 11.83 (s, 2H), 7.42–7.34 (m, 4H), 7.08–7.03 (m, 2H), 7.03 (s, 2H); ^13^C̲ NMR (400 MHz, DMSO-d_6_): 162.94, 158.74, 156.42, 134.39, 130.54, 127.36, 127.25, 114.21, 113.70, 113.44, 107.65, 106.51, 106.43, 106.29, 106.2; HRMS (ESI) *m*/*z* calcd for C_18_H_10_F_2_N_4_Se (M + H)^+^ 401.0111, found 401.0097; elemental analysis: C, 54.15; H, 2.52; F, 9.52; N, 14.03; Se, 19.78, found: C, 54.21; H, 2.48; F, 9.53; N, 14.06; Se, 19.72.

## Conflicts of interest

The authors declare no conflicts of interest.

## Supplementary Material

RA-011-D0RA10576G-s001
