# Supplementary material for: I2/DMSO-catalyzed one-pot approach for the synthesis of 1,3,4-selenadiazoles
Source: RSC Adv. 2021 Feb 2;11(10):5724–8. doi: 10.1039/d0ra10576g (PMC8694770; doi:10.1039/d0ra10576g)
Supplement: RA-011-D0RA10576G-s001 [file RA-011-D0RA10576G-s001.pdf]

Electronic Supplementary Material (ESI) for RSC Advances.
This journal is © The Royal Society of Chemistry 2021

I_2/DMSO-catalyzed one-pot approach for the synthesis of 1,3,4-Selenadiazoles

Suresh Kuarm Bowroju [a], and Rajitha Bavanthula [a]*

Solvents were dried over standard drying agents and were freshly distilled prior to use. Chemicals were purchased from Aldrich and Fluka firms and used without further purification. All column chromatographic separations were performed using silica gel (Acme's, 60–120 mesh). Organic solutions were dried over anhydrous Na_2SO_4 and concentrated below 40°C in vacuo. Melting points are uncorrected and were determined in open capillaries. The reactions were monitored by thin layer chromatography (TLC) and visualized with UV light. the 1H NMR spectra were recorded on Bruker Avance 400 MHz spectrometer using TMS as an internal standard. Spectral analyses were carried out in $CDCl_3$ or DMSO-d_6 for both ^{1}H and ^{13}C spectra. Chemical shifts were measured in δ parts per million (*ppm*) and coupling constants (*J*) were measured in hertz (Hz). HRMS spectra were recorded on XEVO G2-XS QTOF Spectrometer. The elemental analysis of the compounds was done on a Carlo Erba Model EA1108.

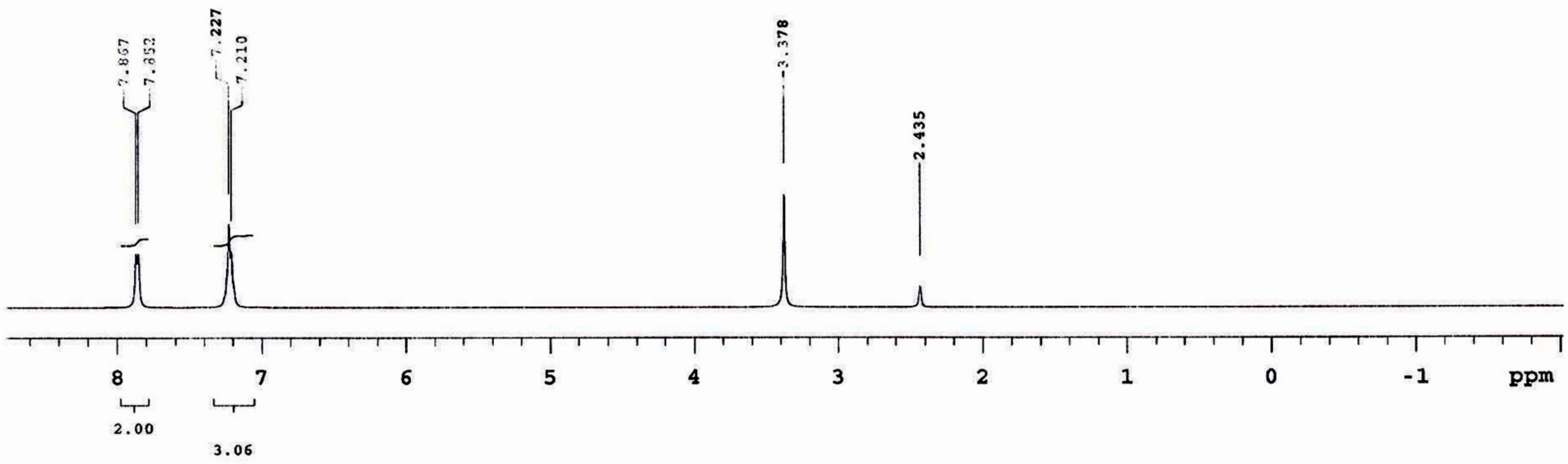

^1H NMR spectrum of compound 3a in DMSO-d_6

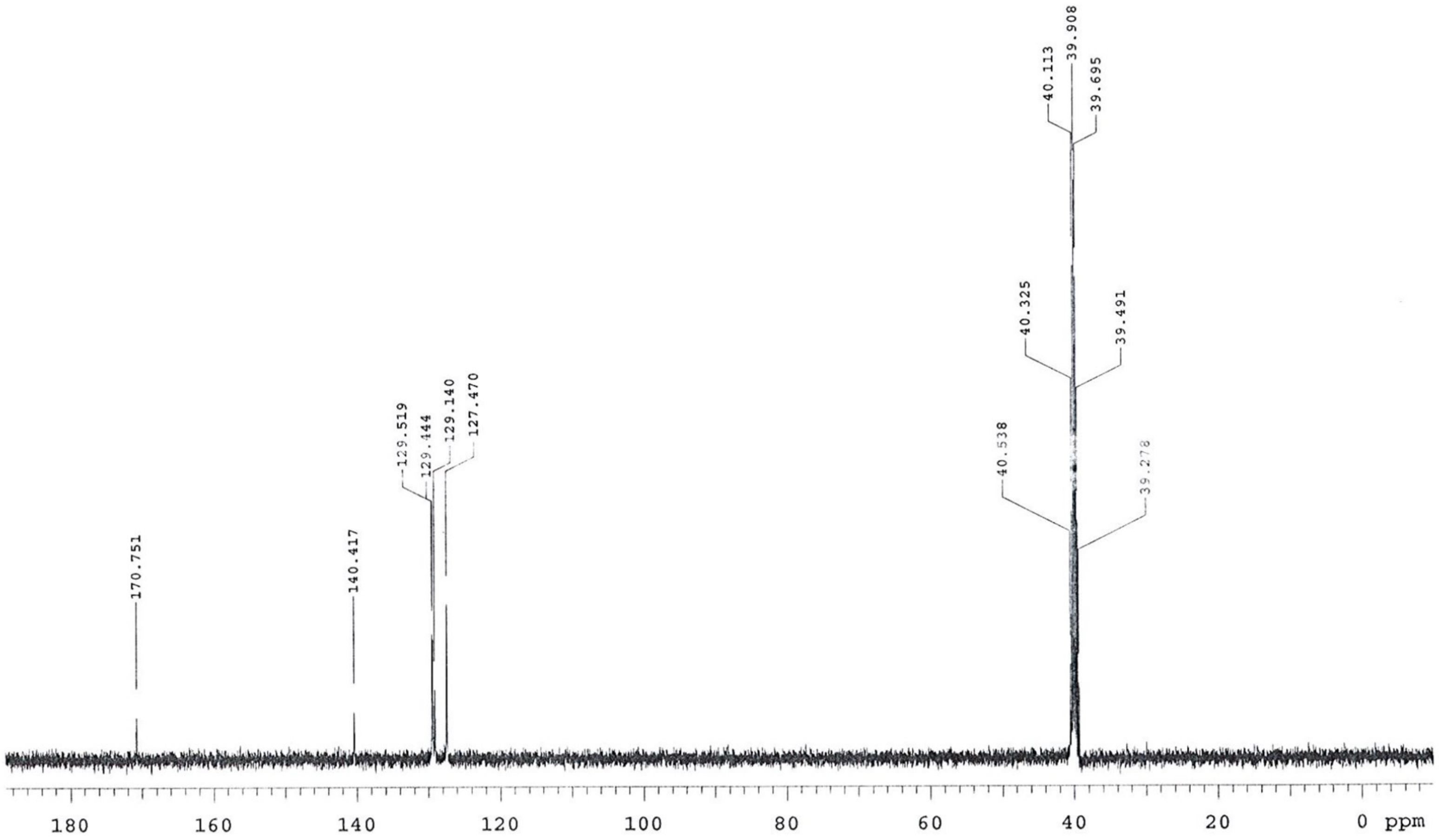

^{13}C NMR spectrum of compound 3a in DMSO-d_6

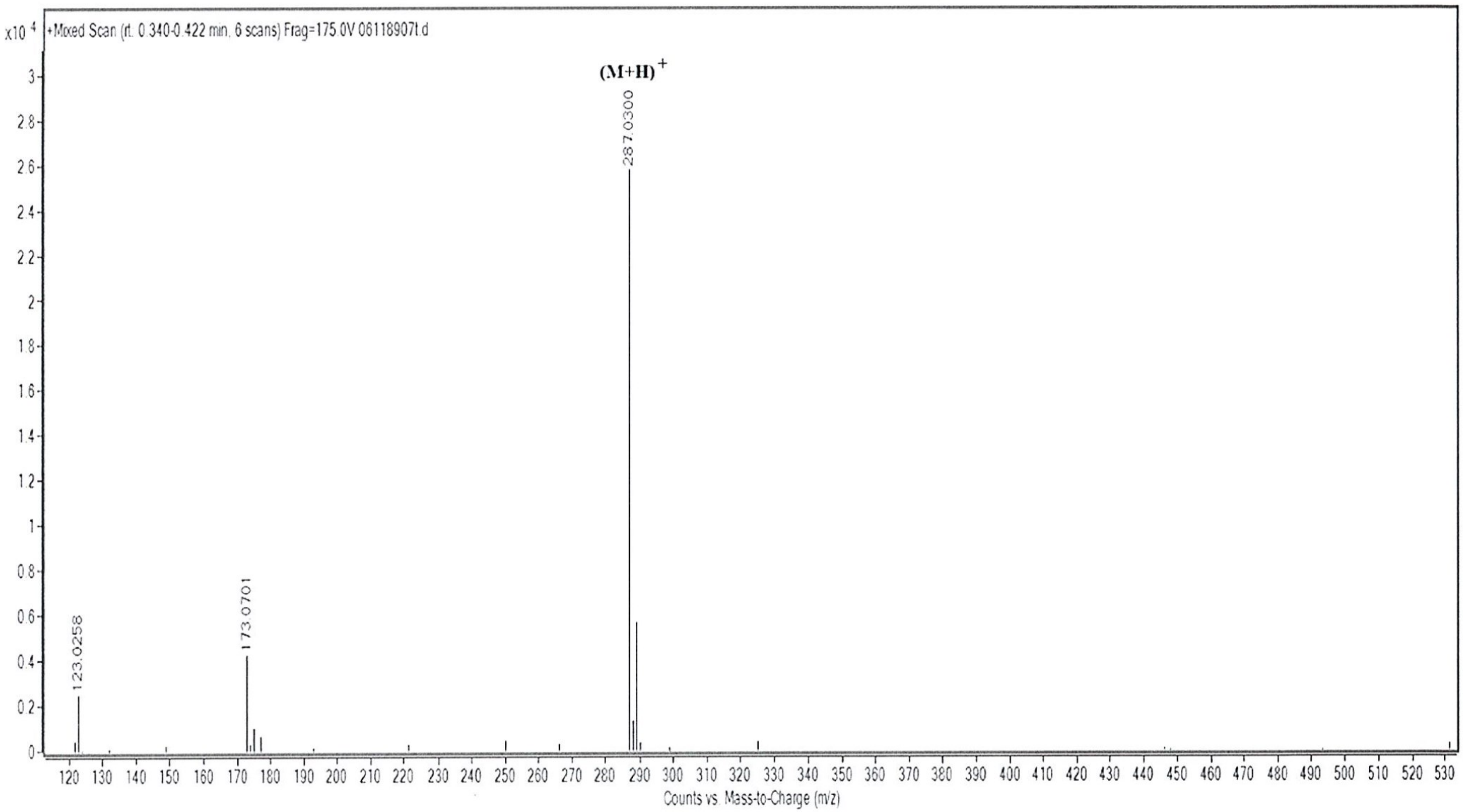

HRMS spectrum of compound 3a

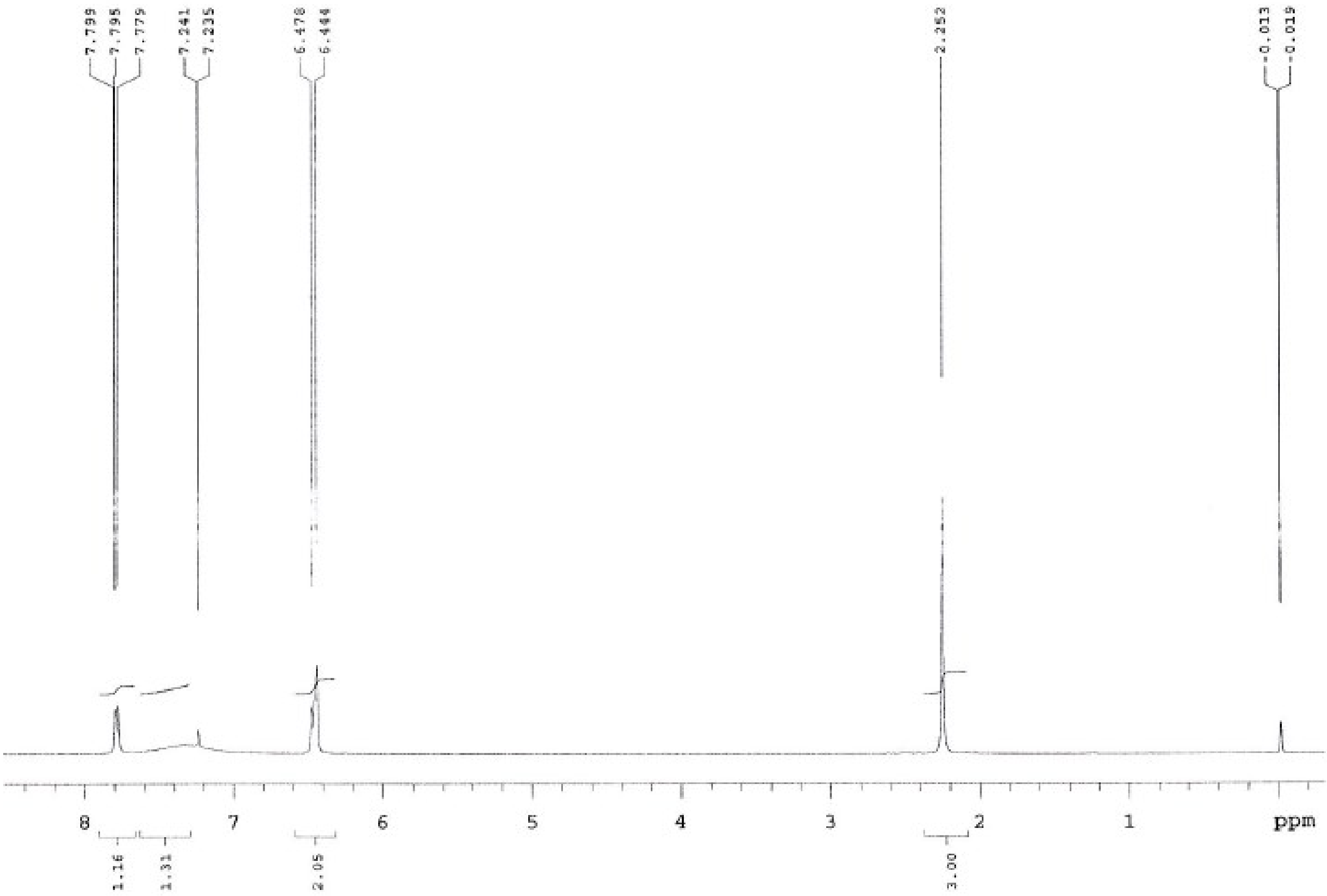

^{1}H NMR spectrum of compound **3b** in $CDCl_3$

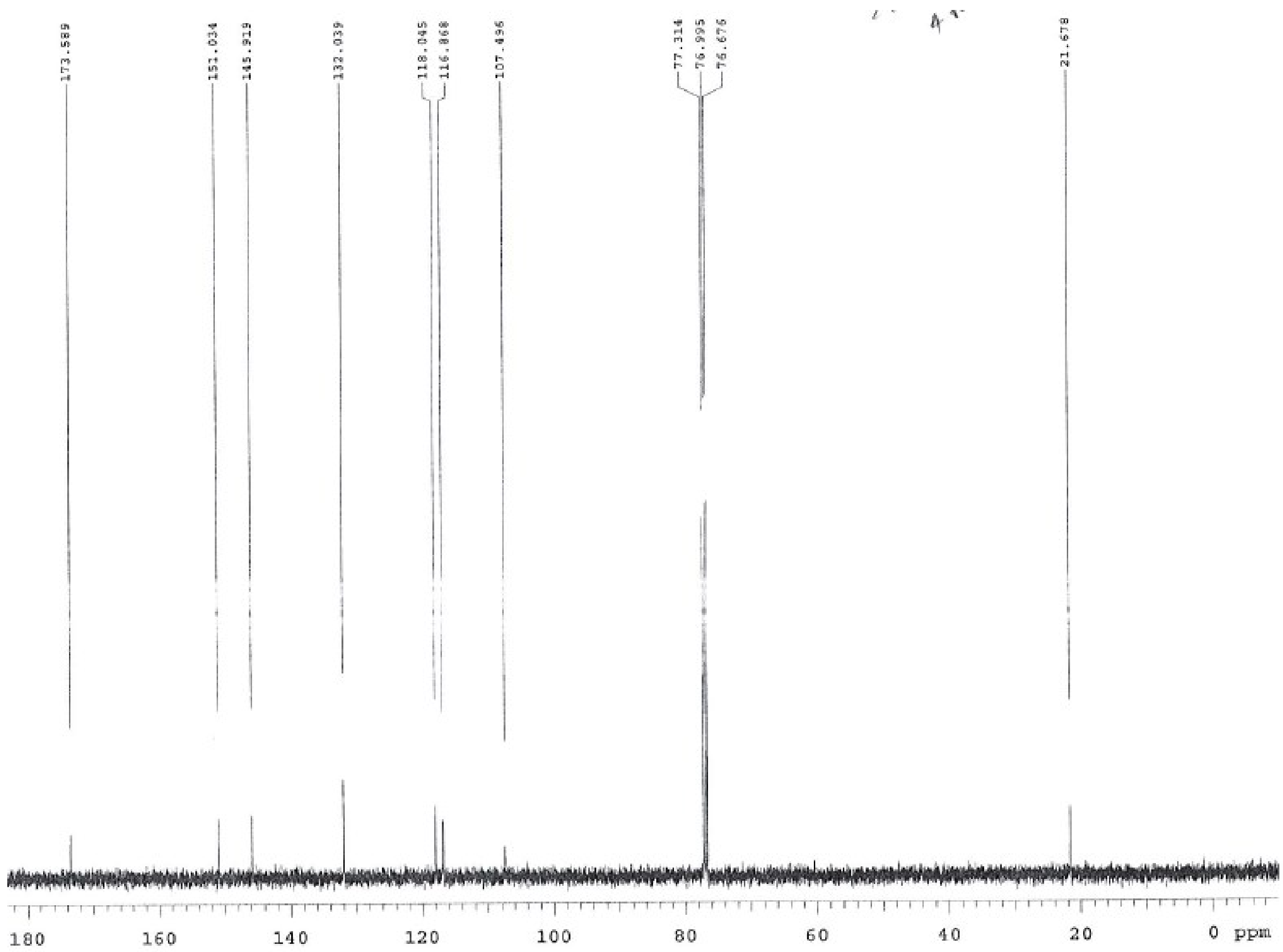

^{13}C NMR spectrum of compound 3b in $CDCl_3$

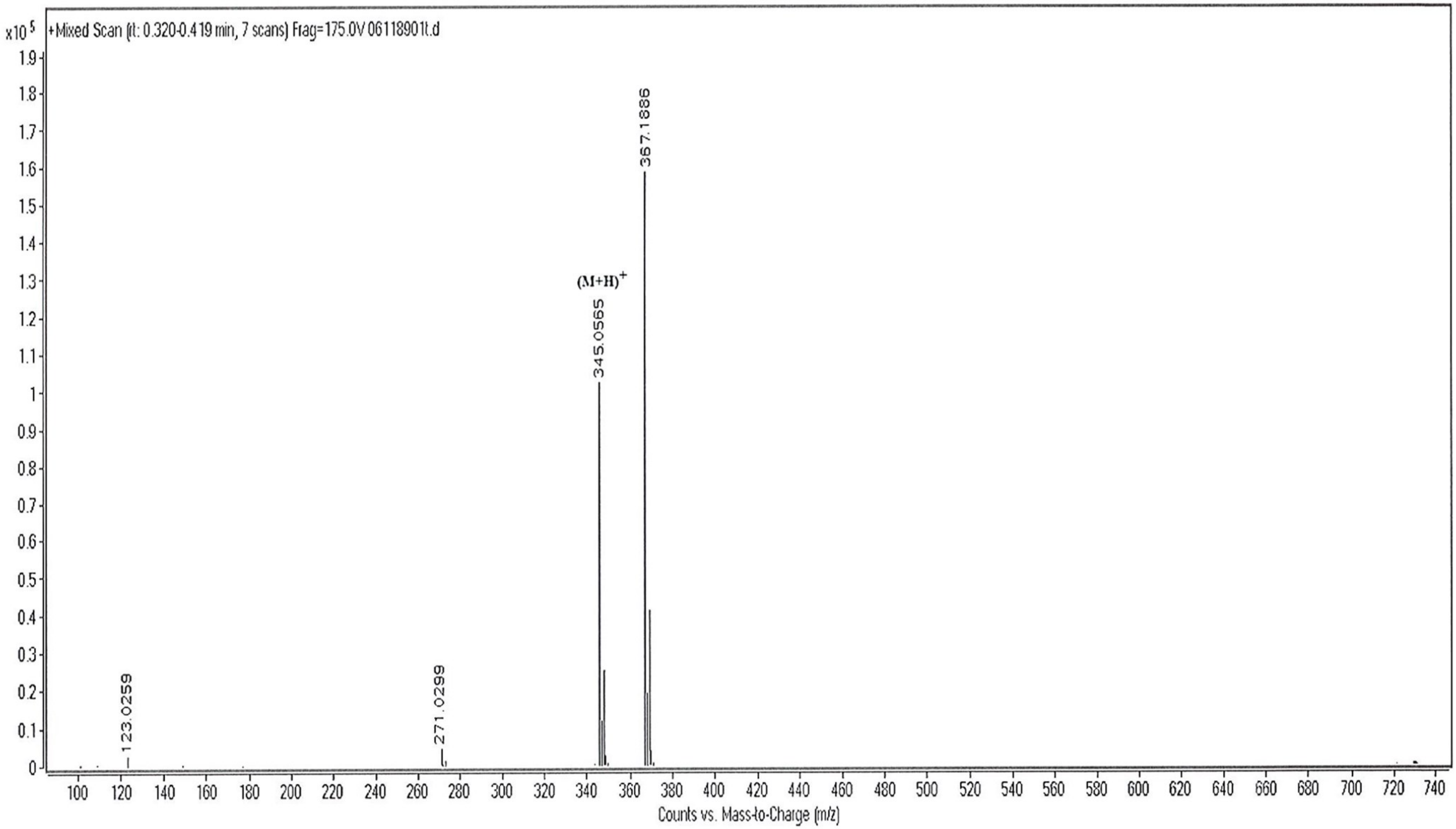

HRMS spectrum of compound 3b

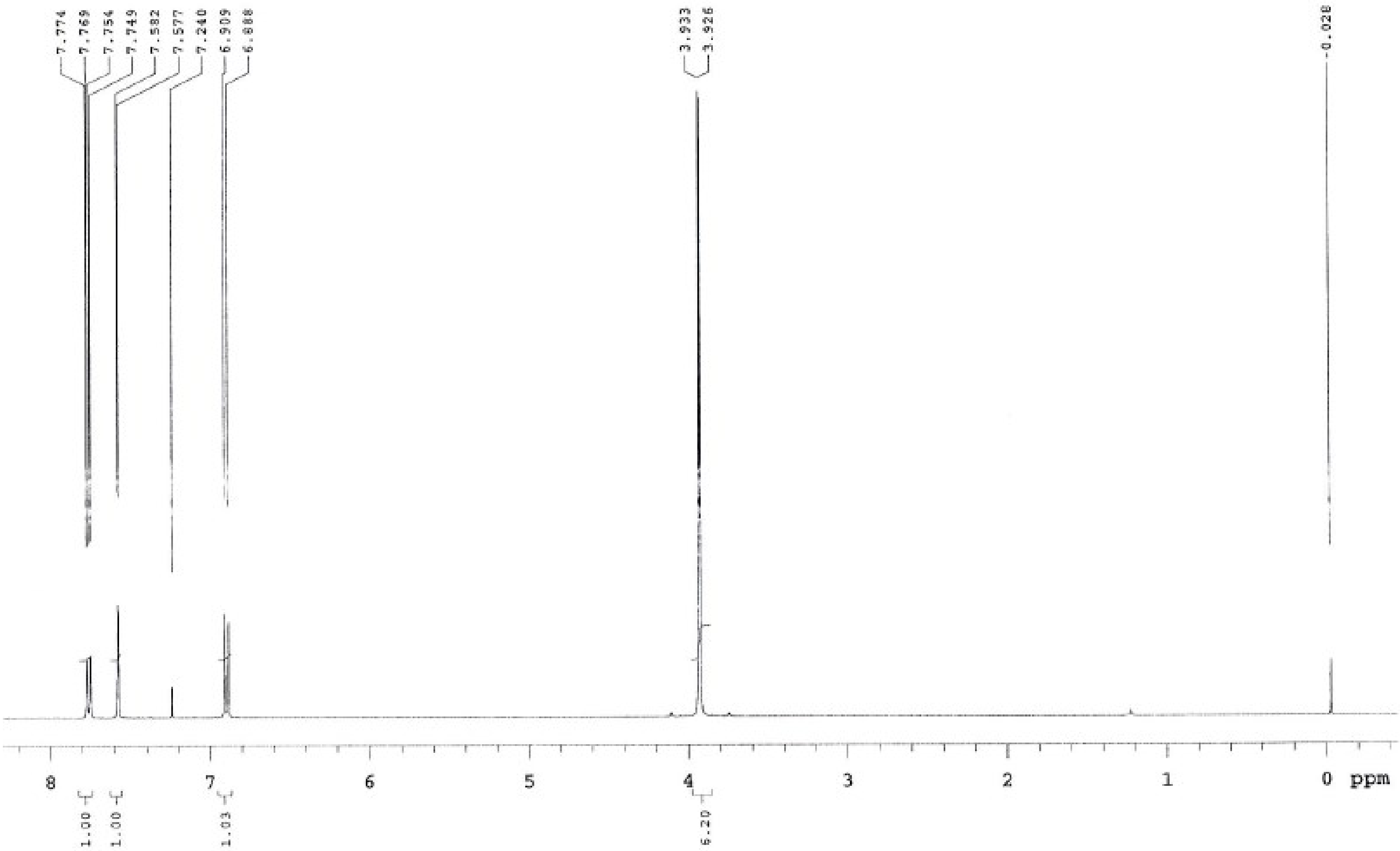

^{1}H NMR spectrum of compound 3c in $CDCl_3$

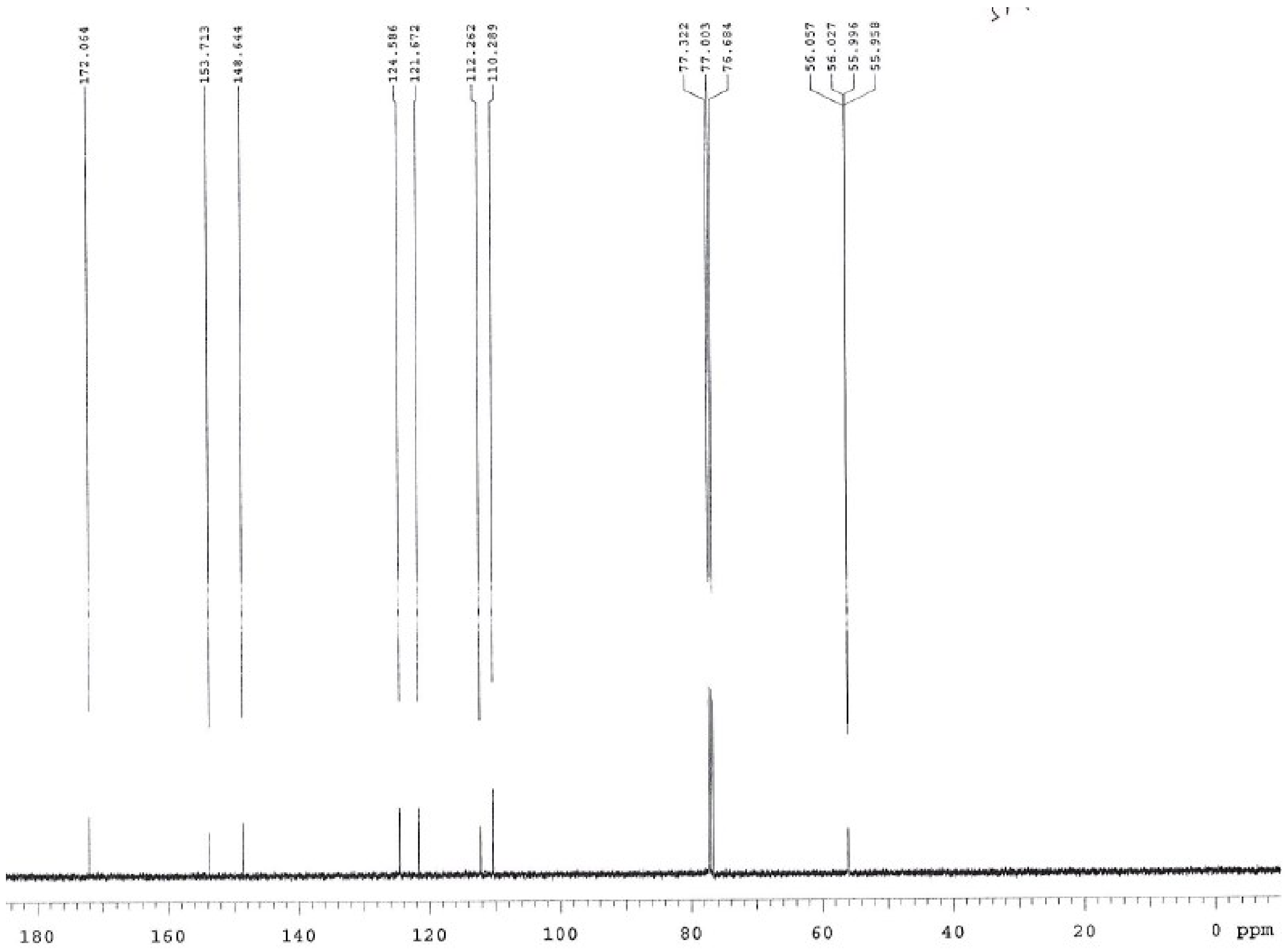

^{13}C NMR spectrum of compound 3c in $CDCl_3$

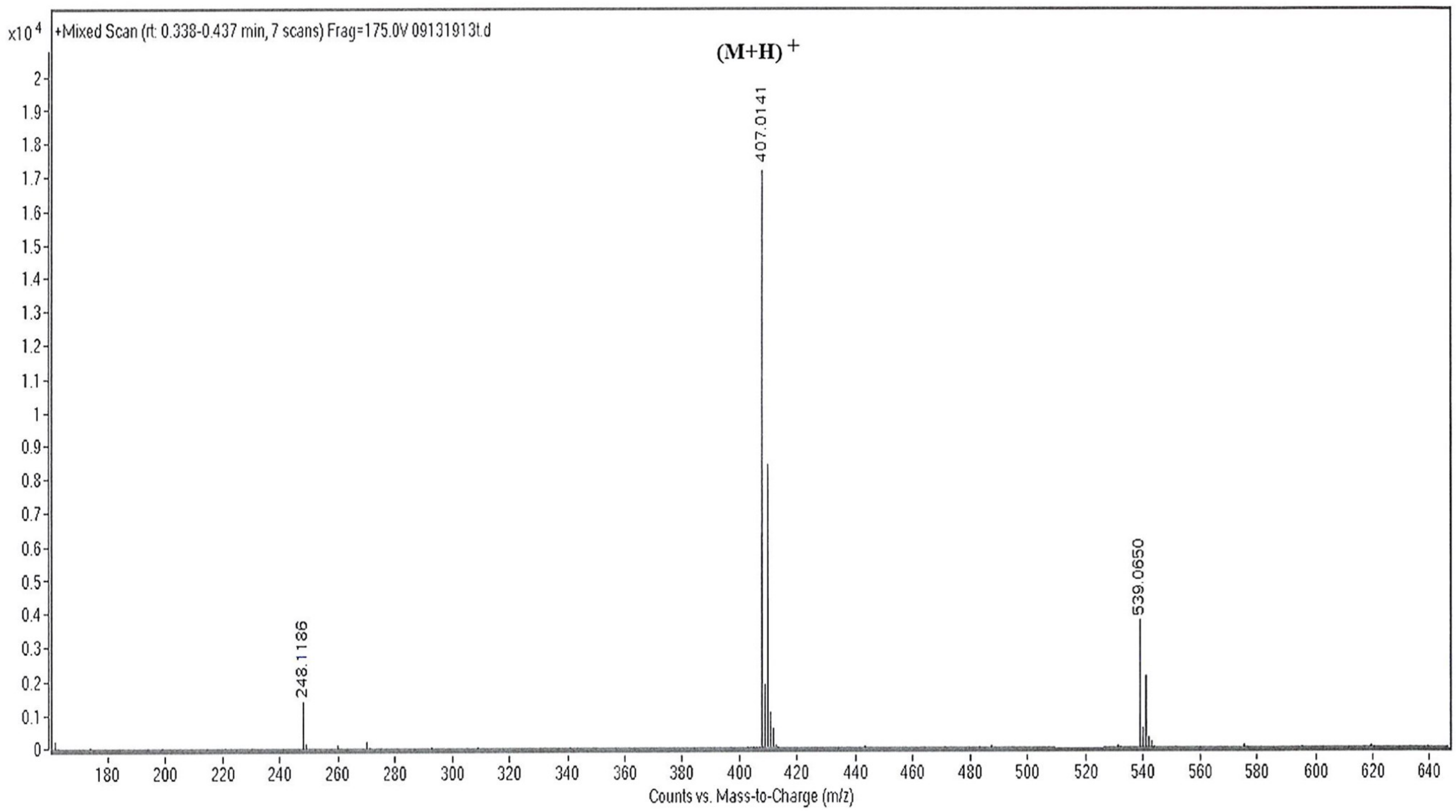

HRMS spectrum of compound 3c

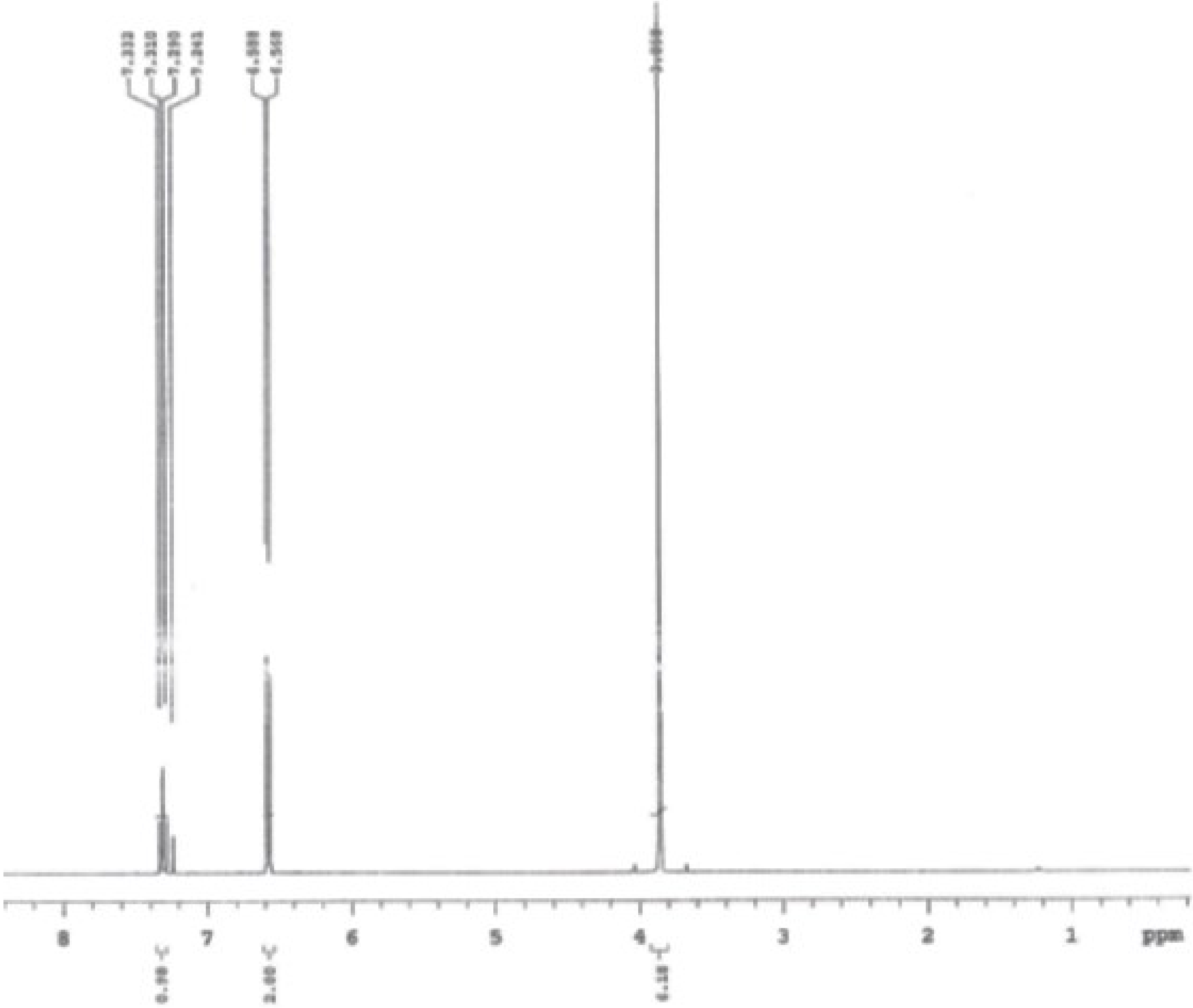

^{1}H NMR spectrum of compound 3d in DMSO-d_6

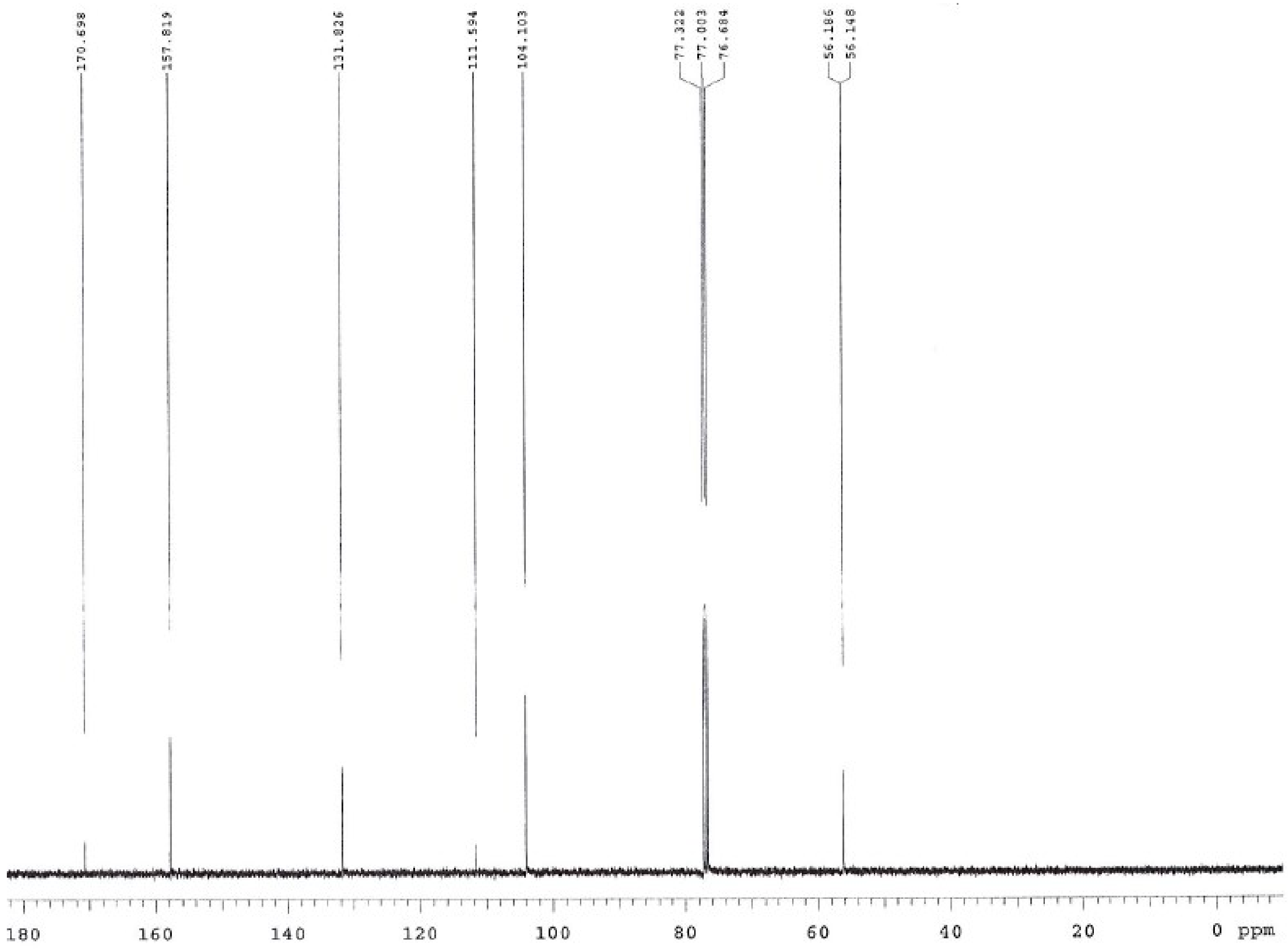

^{13}C NMR spectrum of compound 3d in DMSO-d_6

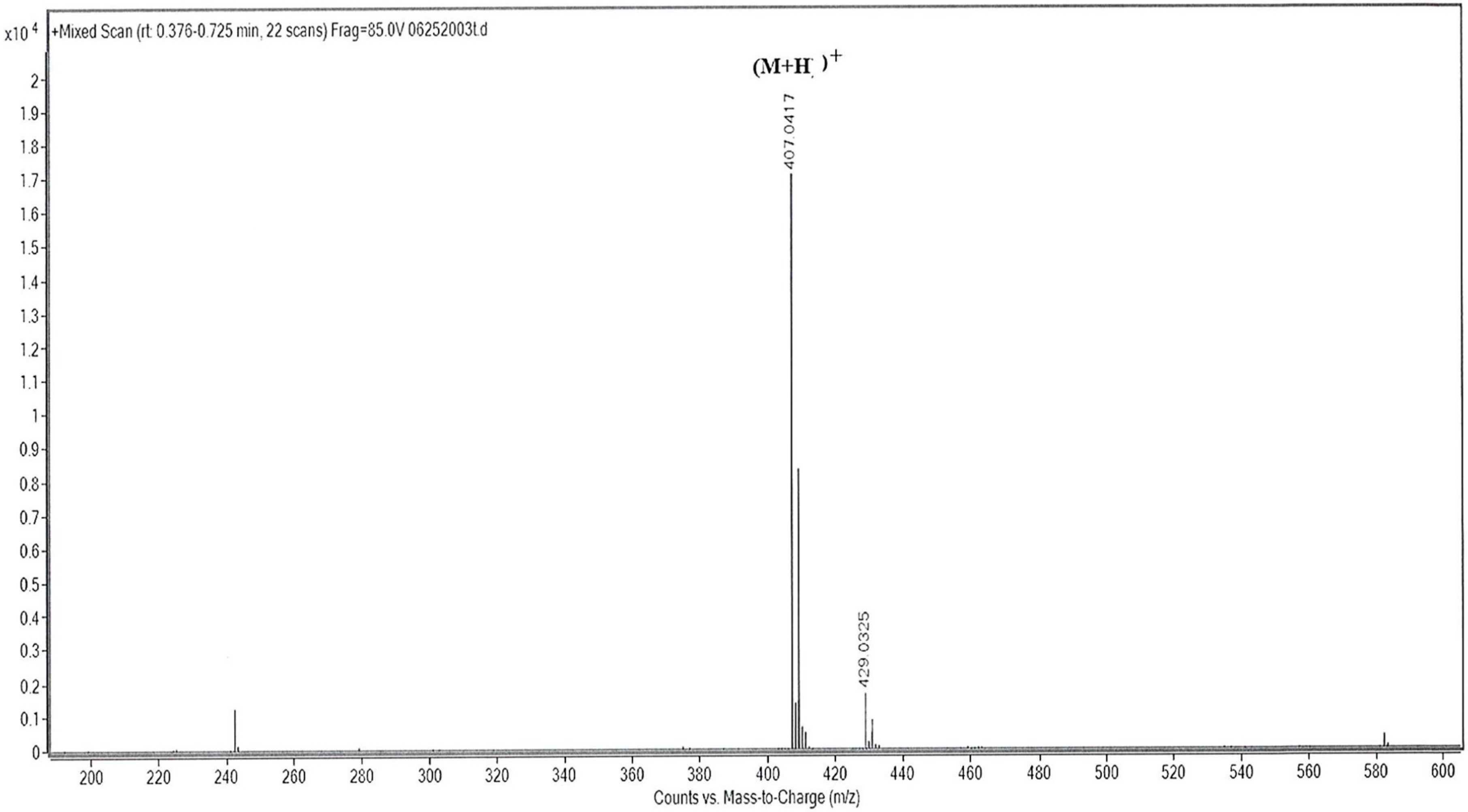

HRMS spectrum of compound 3d

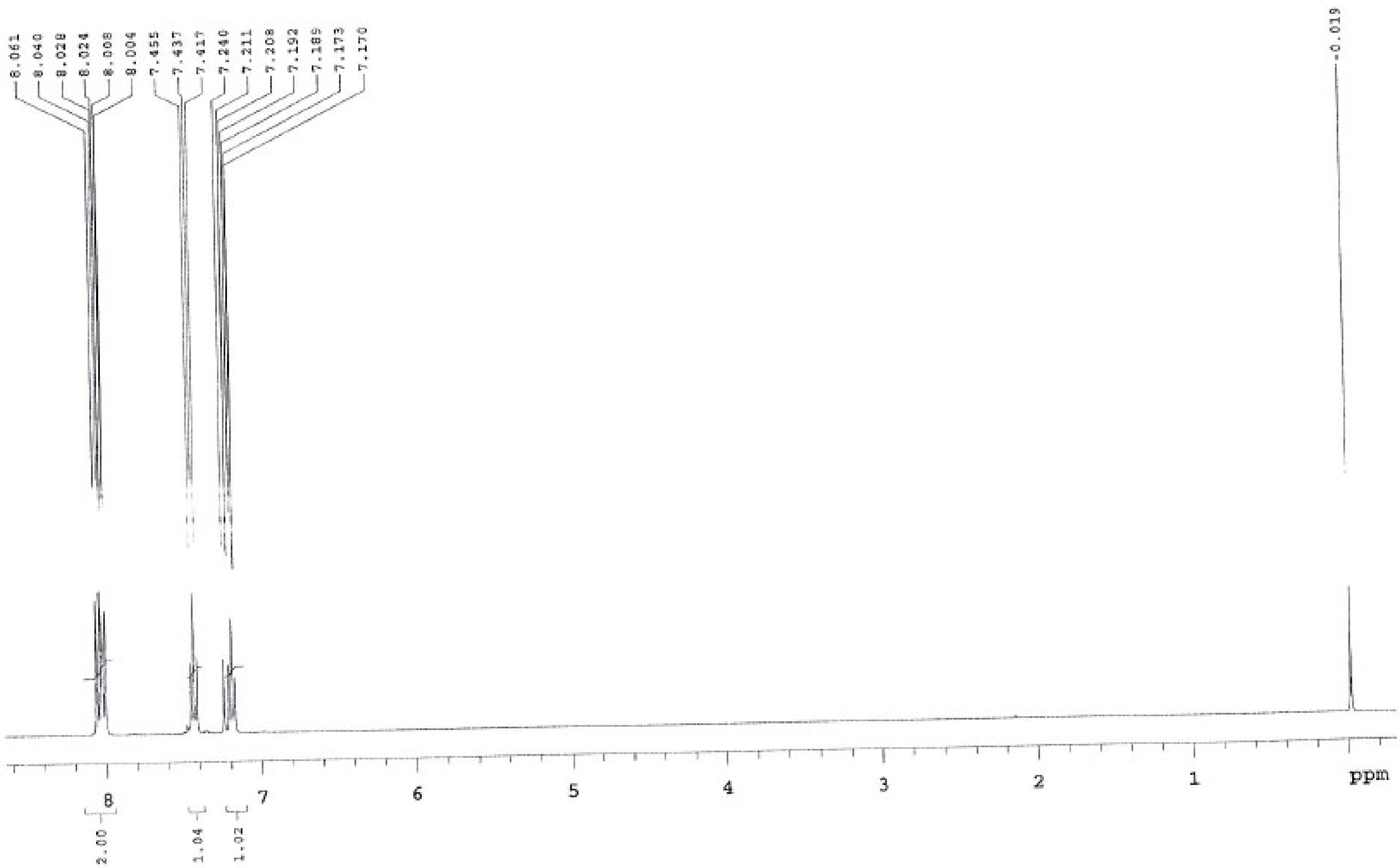

^{1}H NMR spectrum of compound 3e in DMSO-d_6

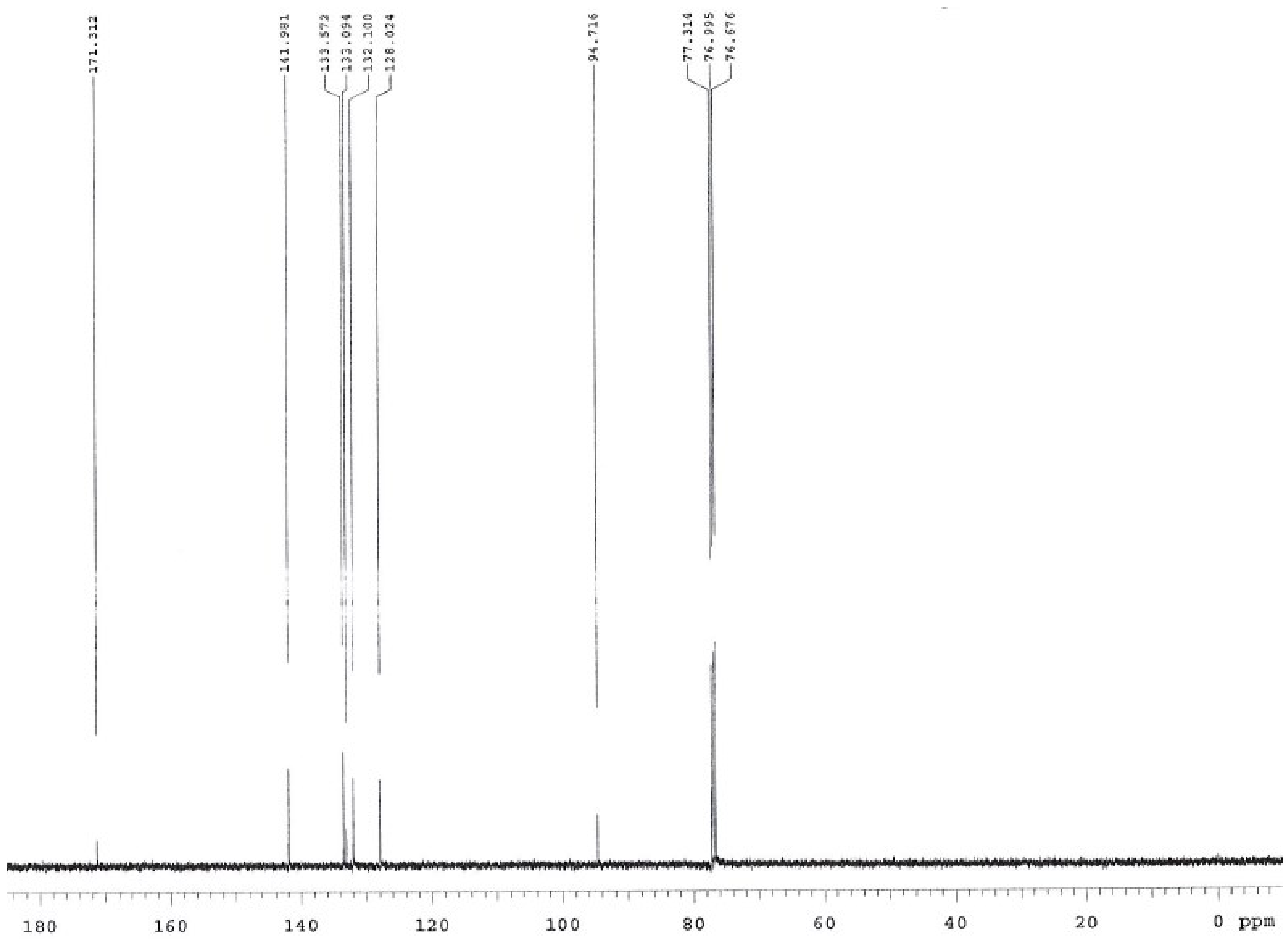

^{13}C NMR spectrum of compound 3e in DMSO-d_6

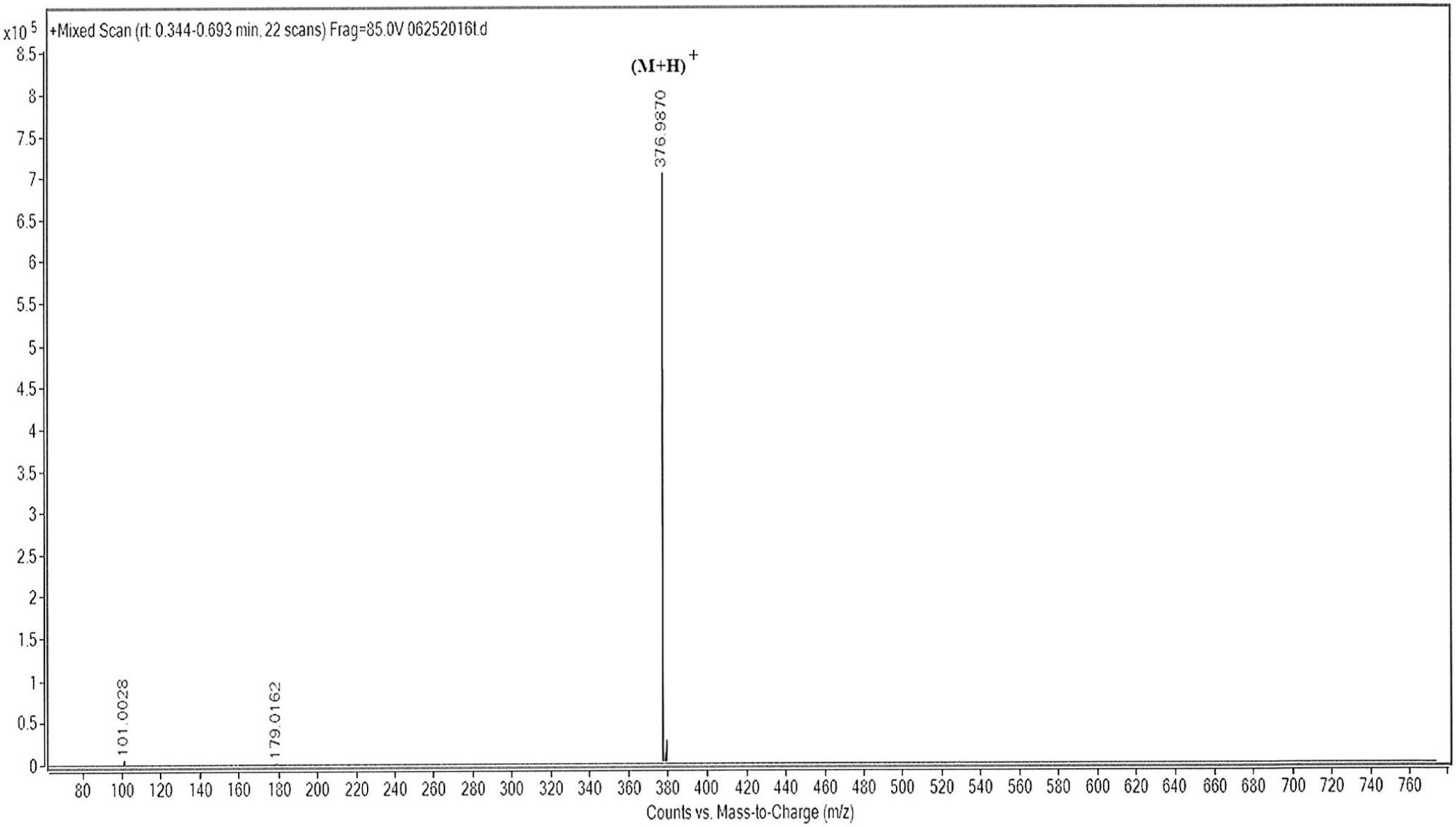

HRMS spectrum of compound 3e

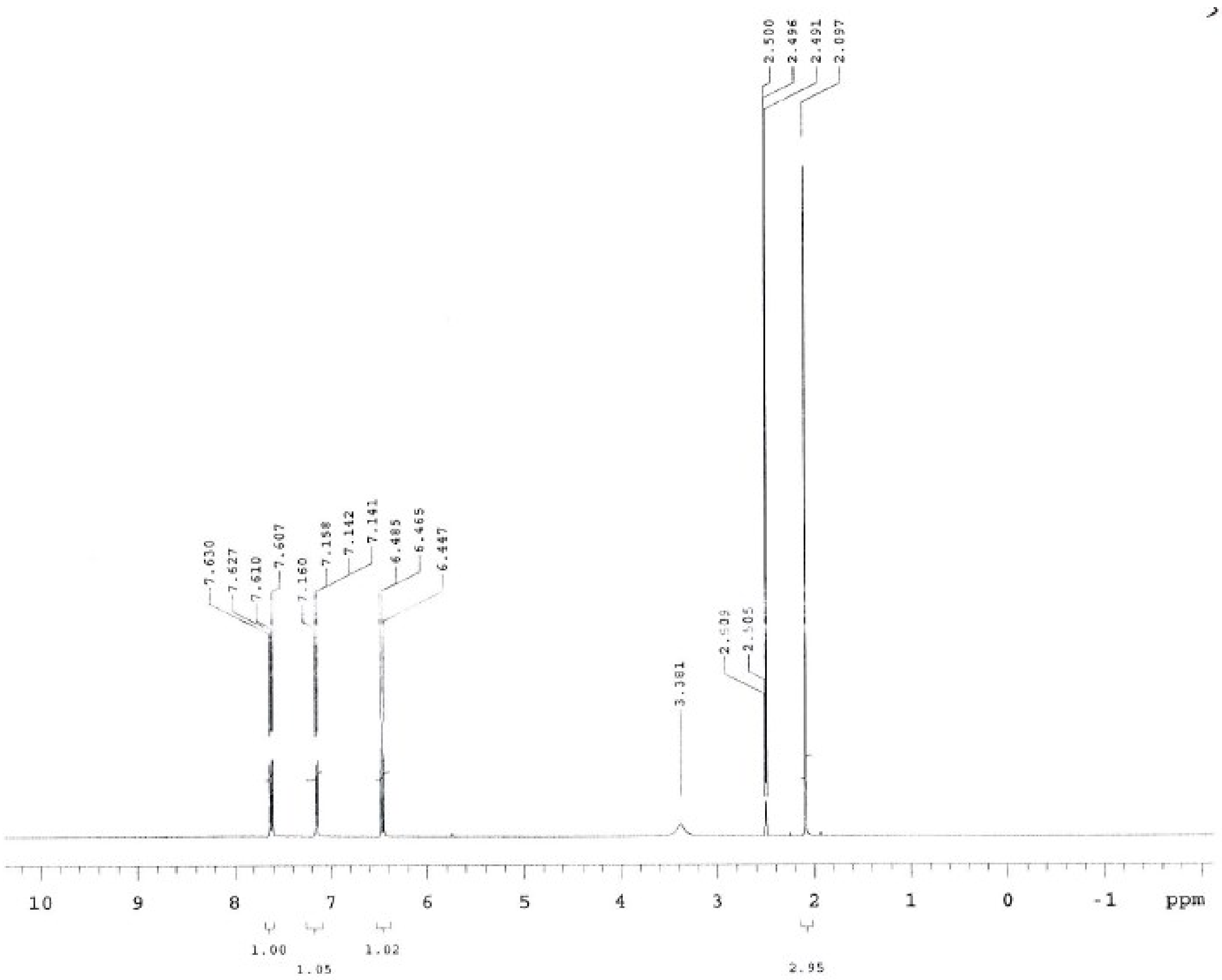

^1H NMR spectrum of compound 3f in DMSO-d_6

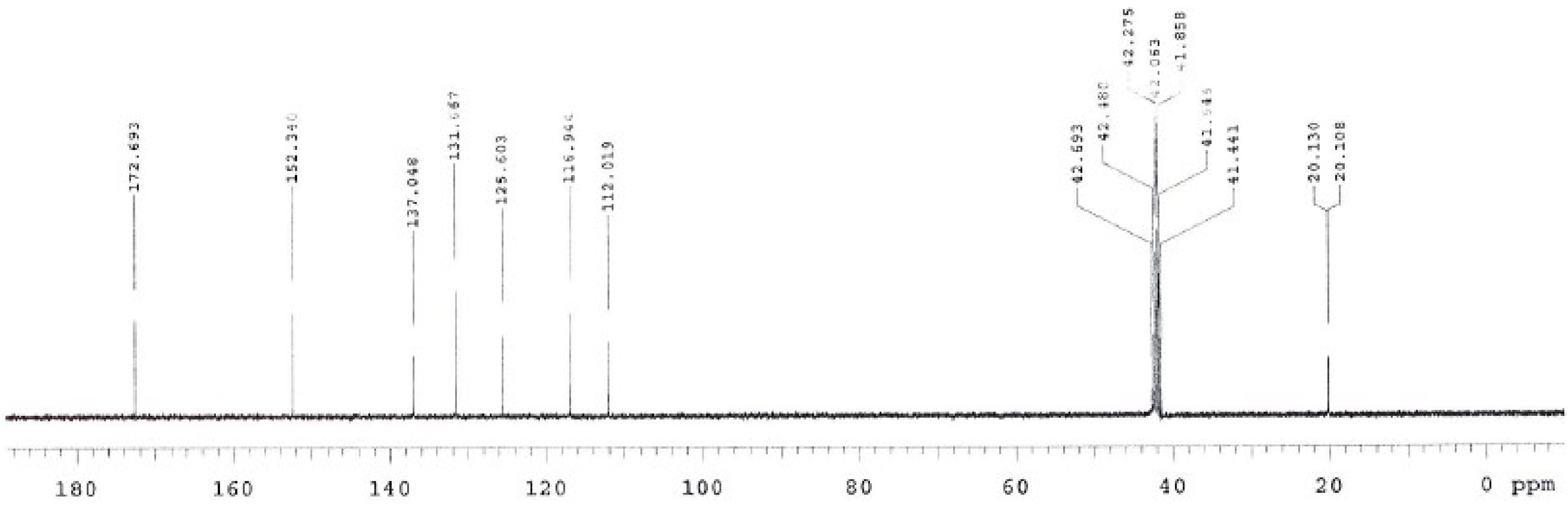

^{13}C NMR spectrum of compound 3f in DMSO-d_6

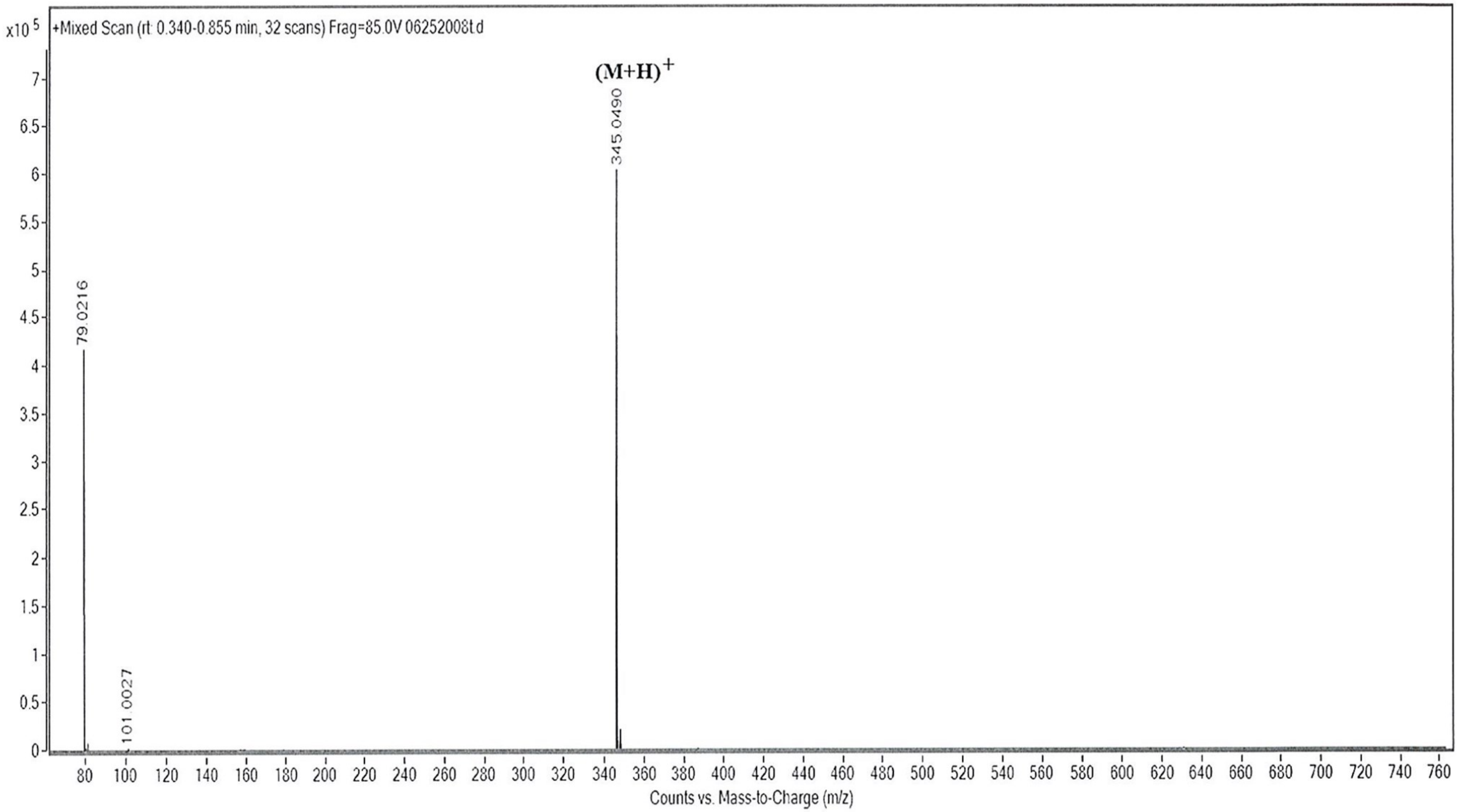

HRMS spectrum of compound 3f

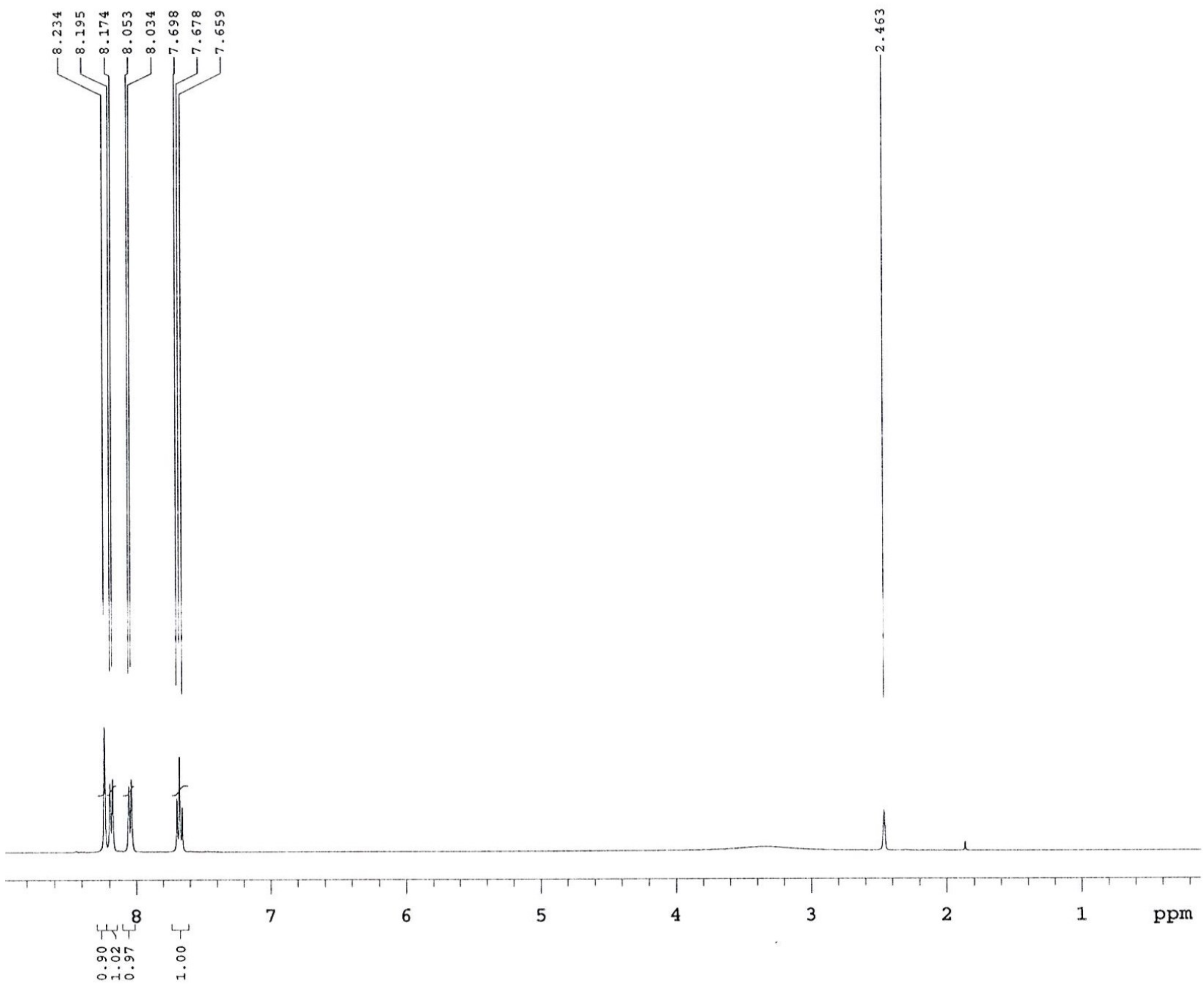

^{1}H NMR spectrum of compound 3g in DMSO-d_6

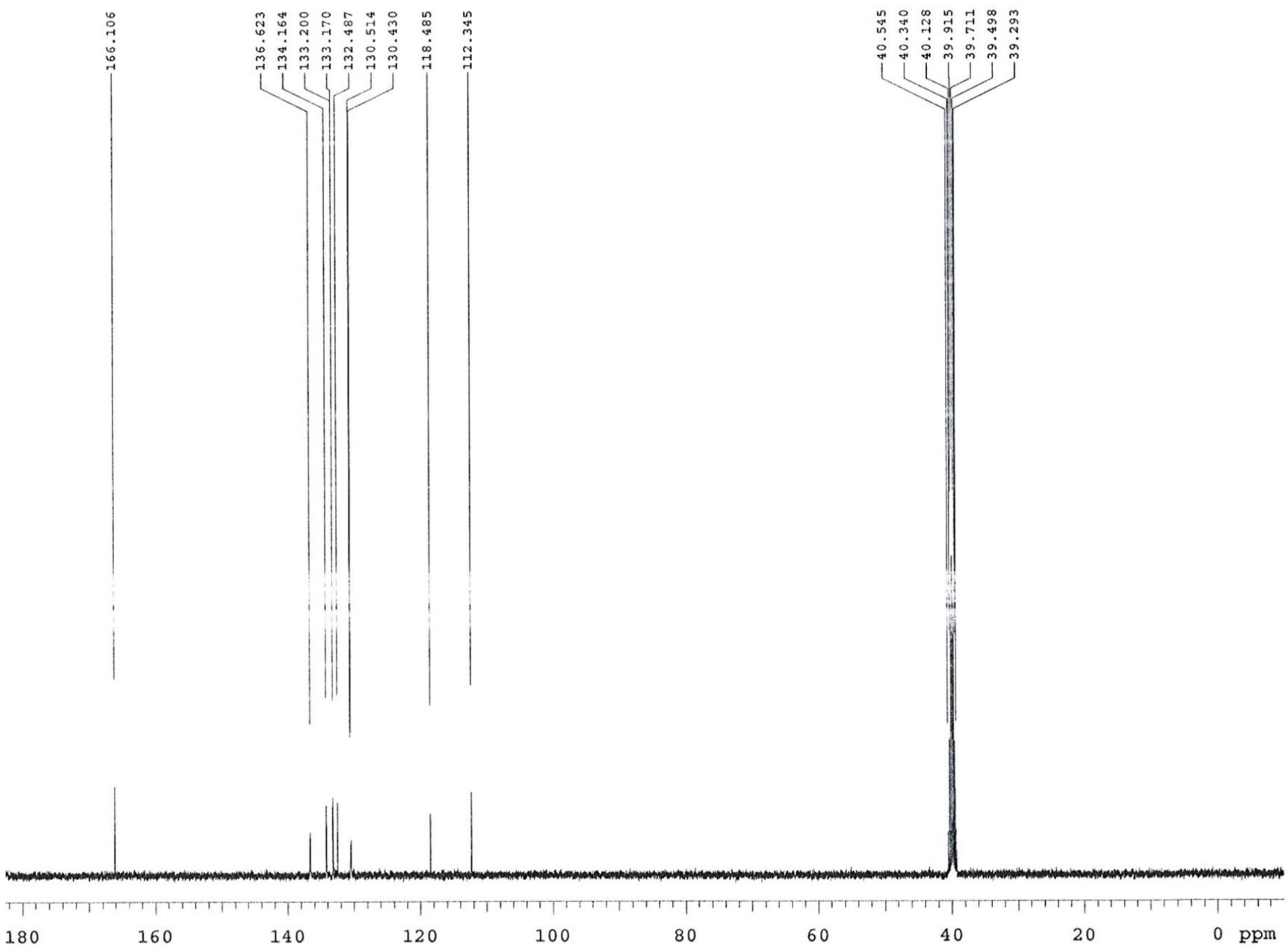

^{13}C NMR spectrum of compound 3g in DMSO-d_6

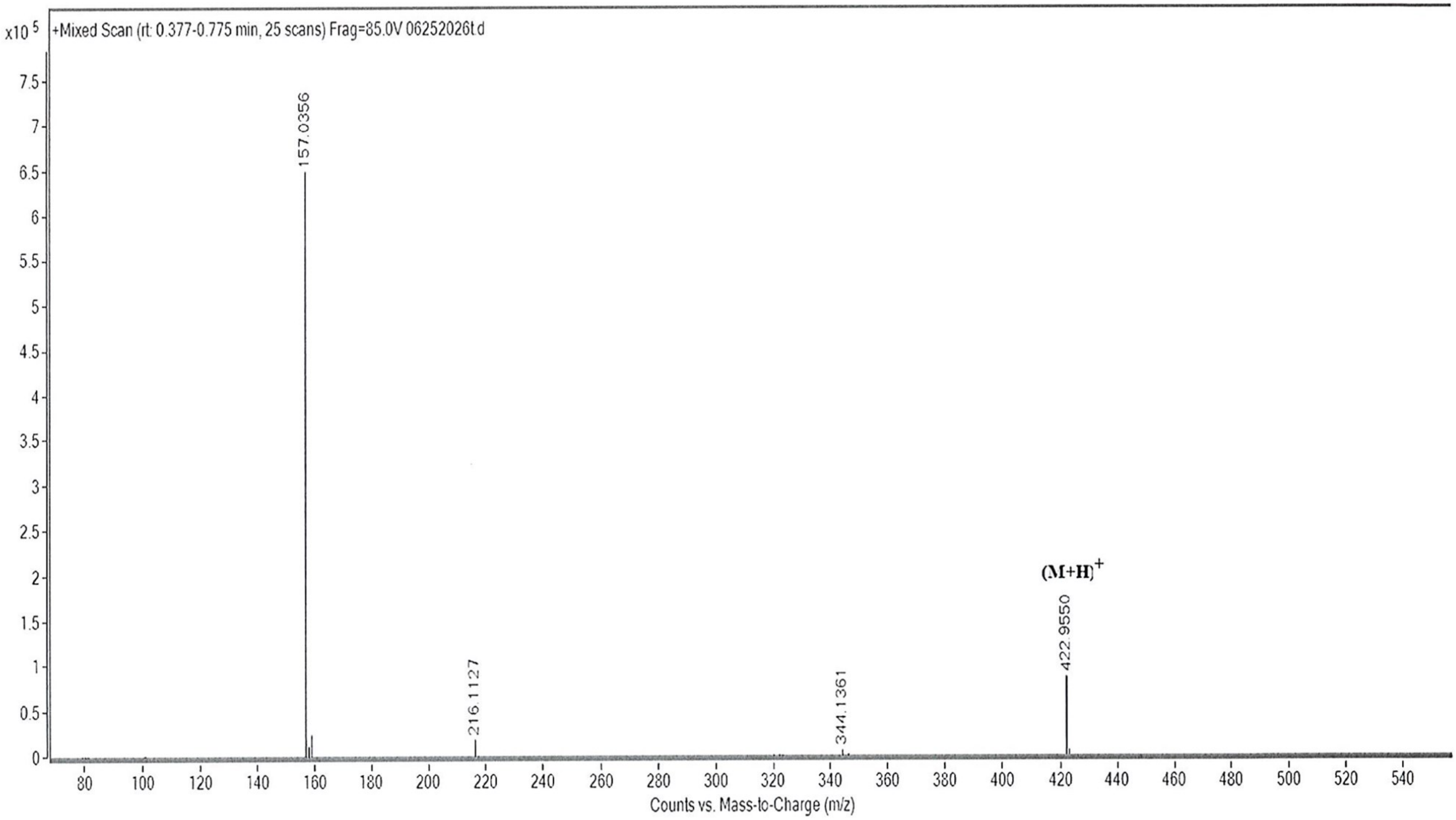

HRMS spectrum of compound 3g

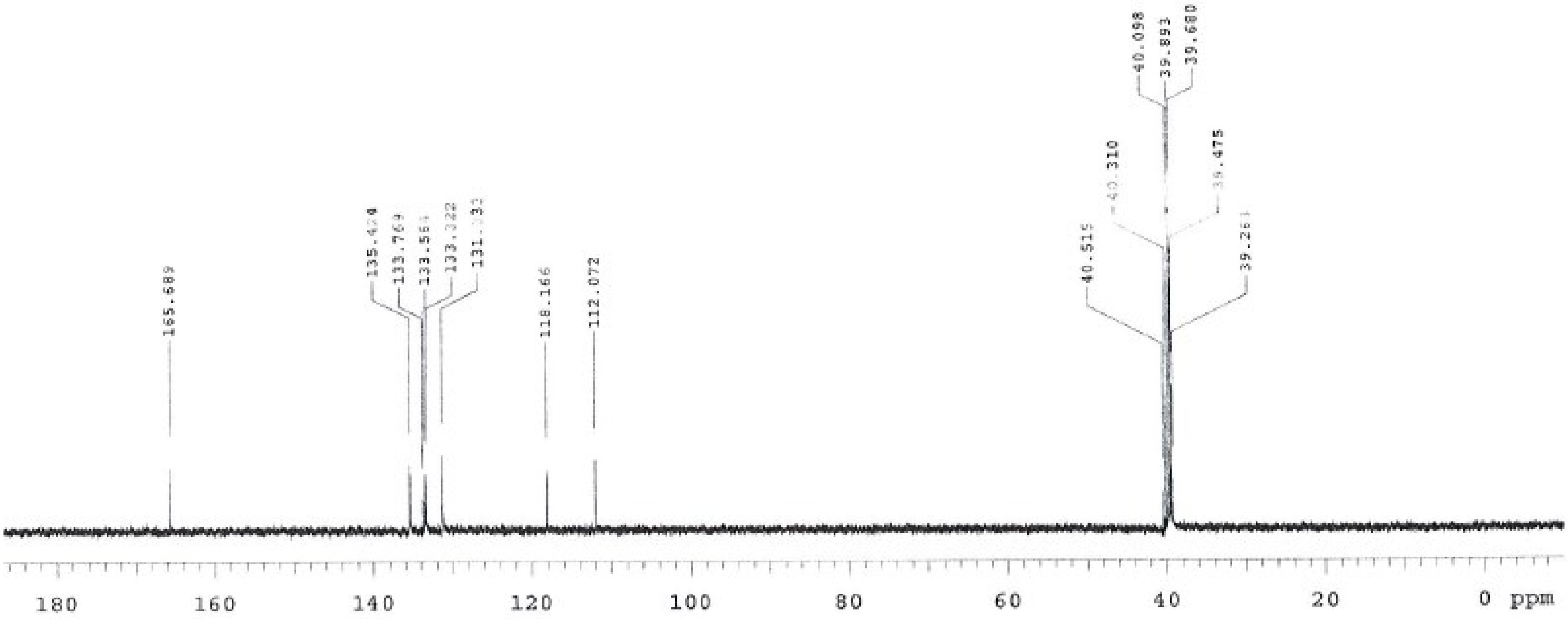

^{1}H NMR spectrum of compound 3h in DMSO-d_6

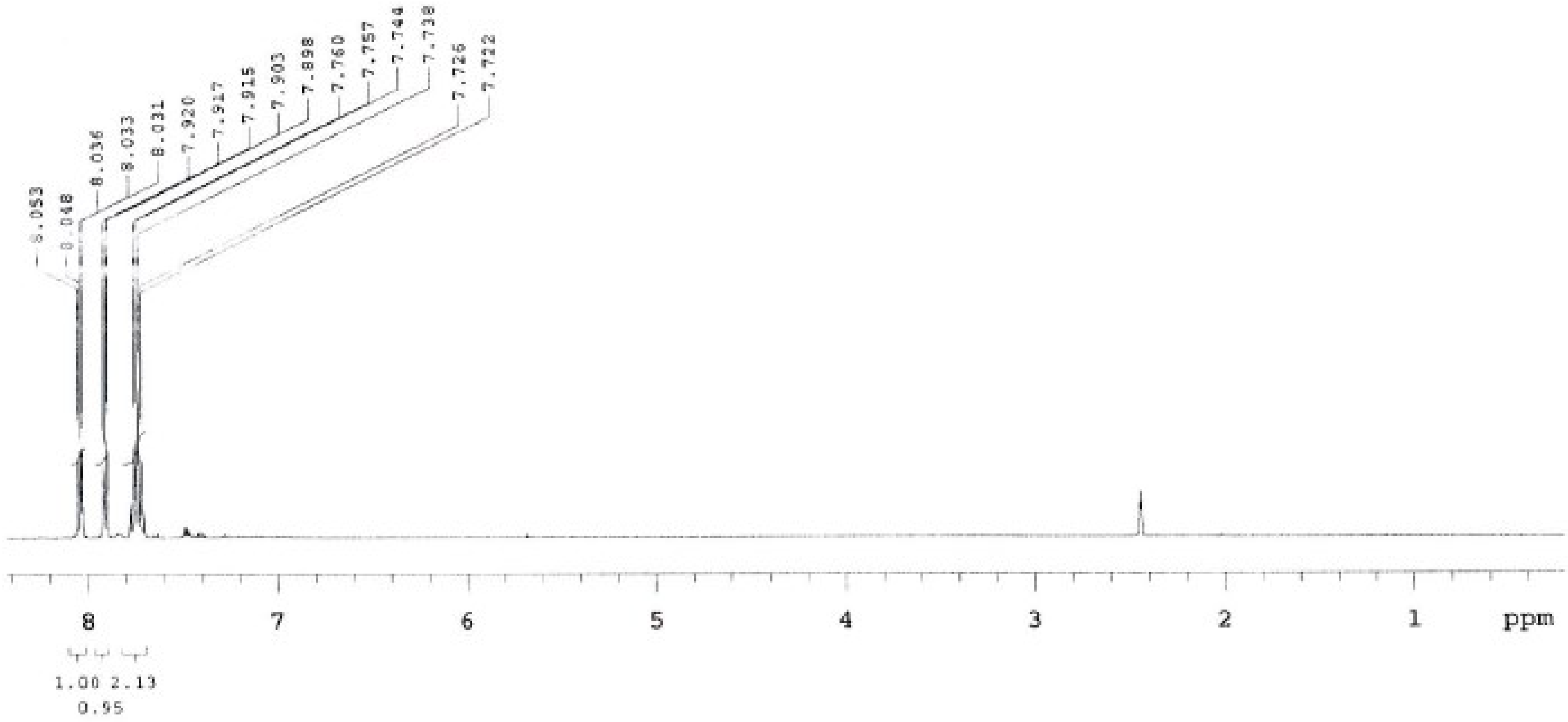

^{13}C NMR spectrum of compound 3h in DMSO-d_6

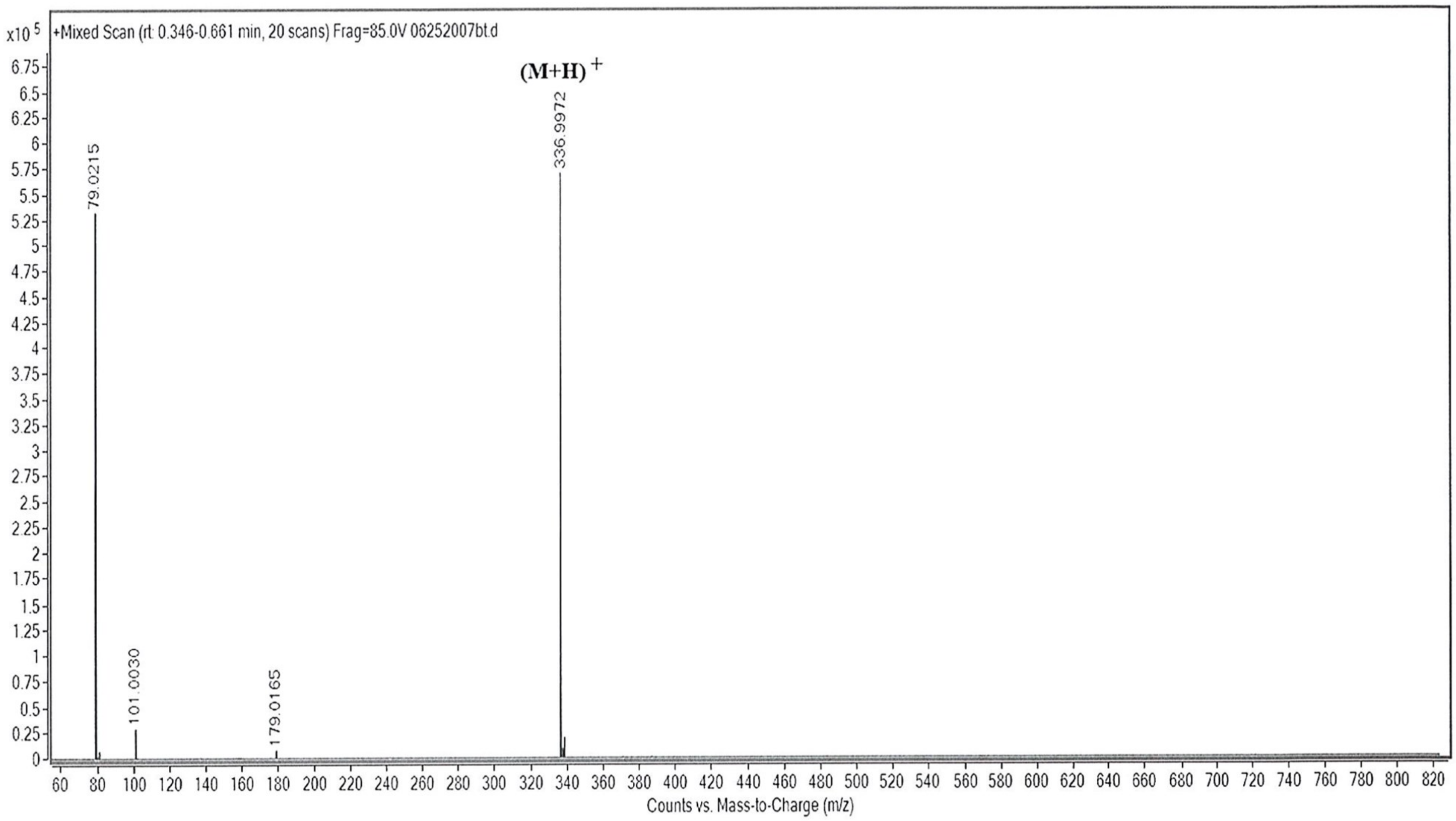

HRMS spectrum of compound 3h

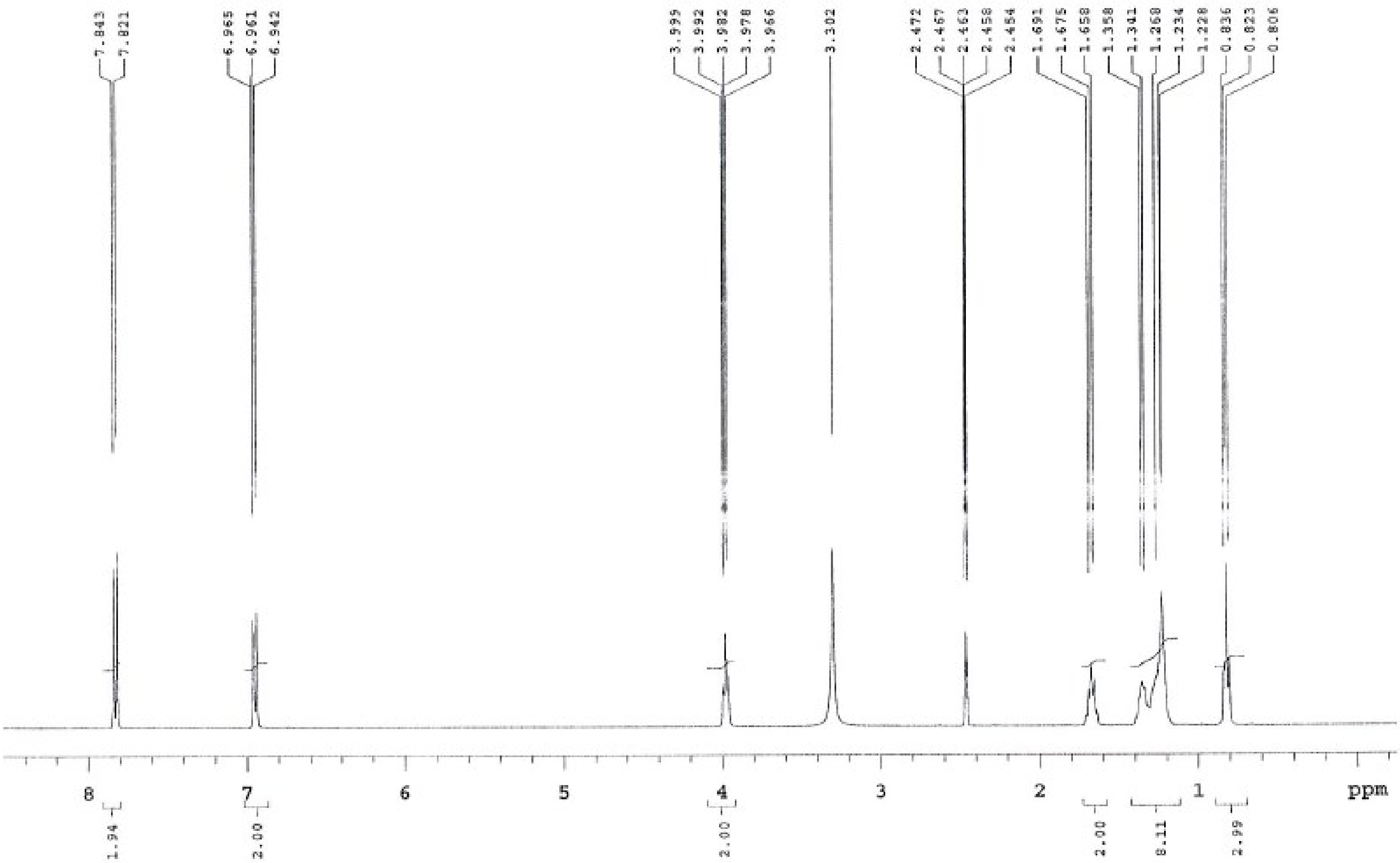

^{1}H NMR spectrum of compound 3i in DMSO-d_6

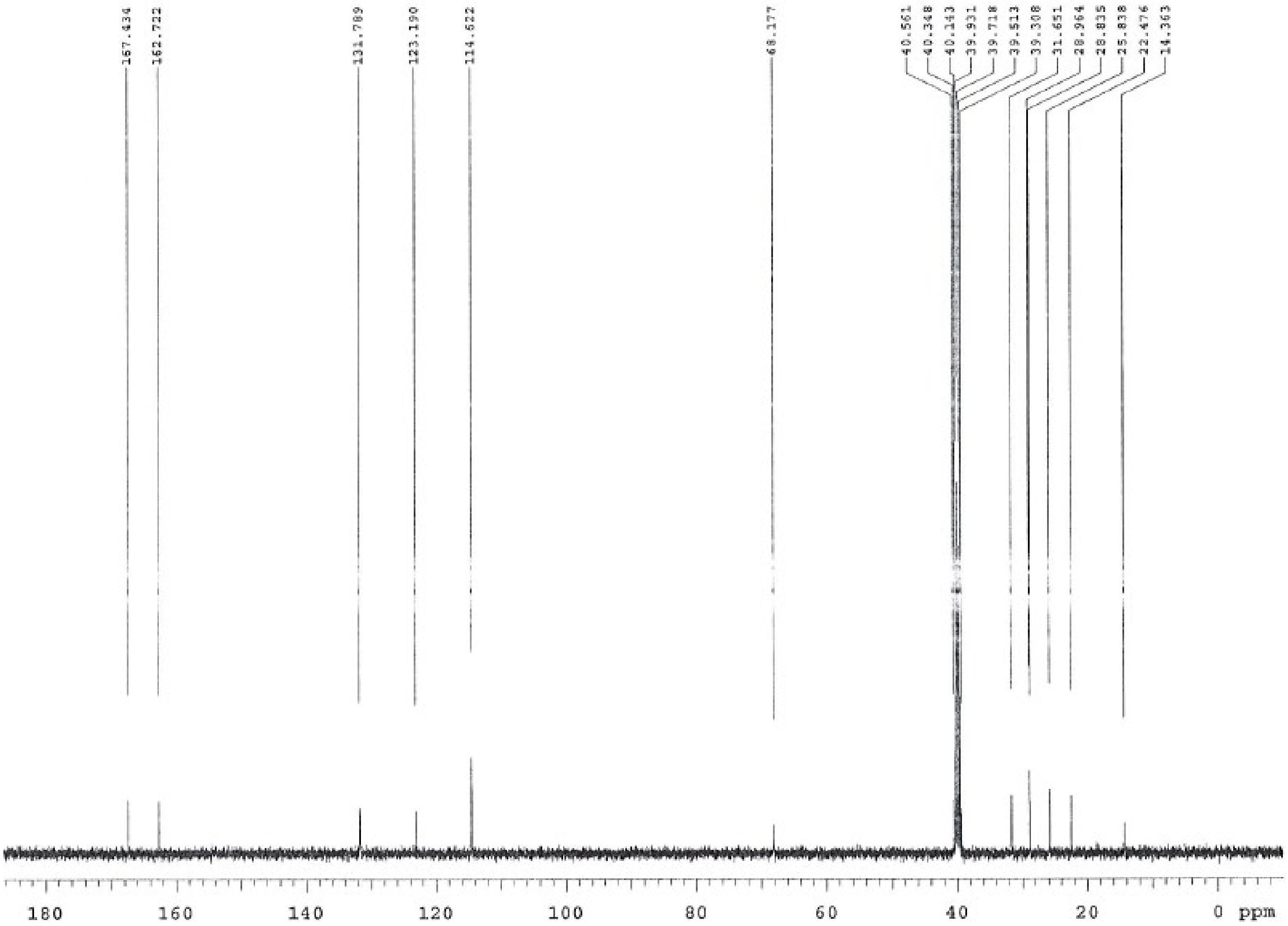

^{13}C NMR spectrum of compound 3i in DMSO-d_6

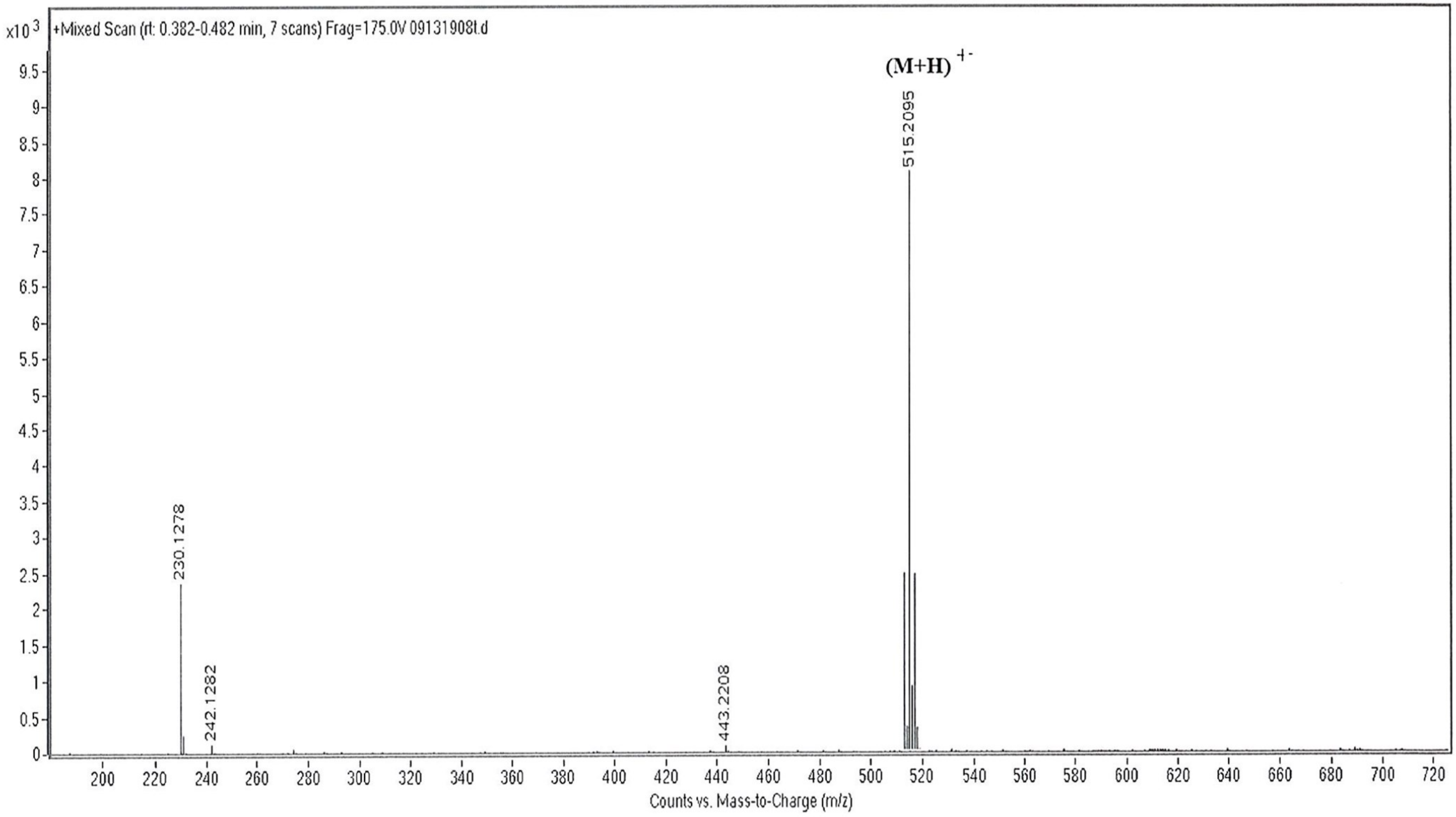

HRMS spectrum of compound 3i

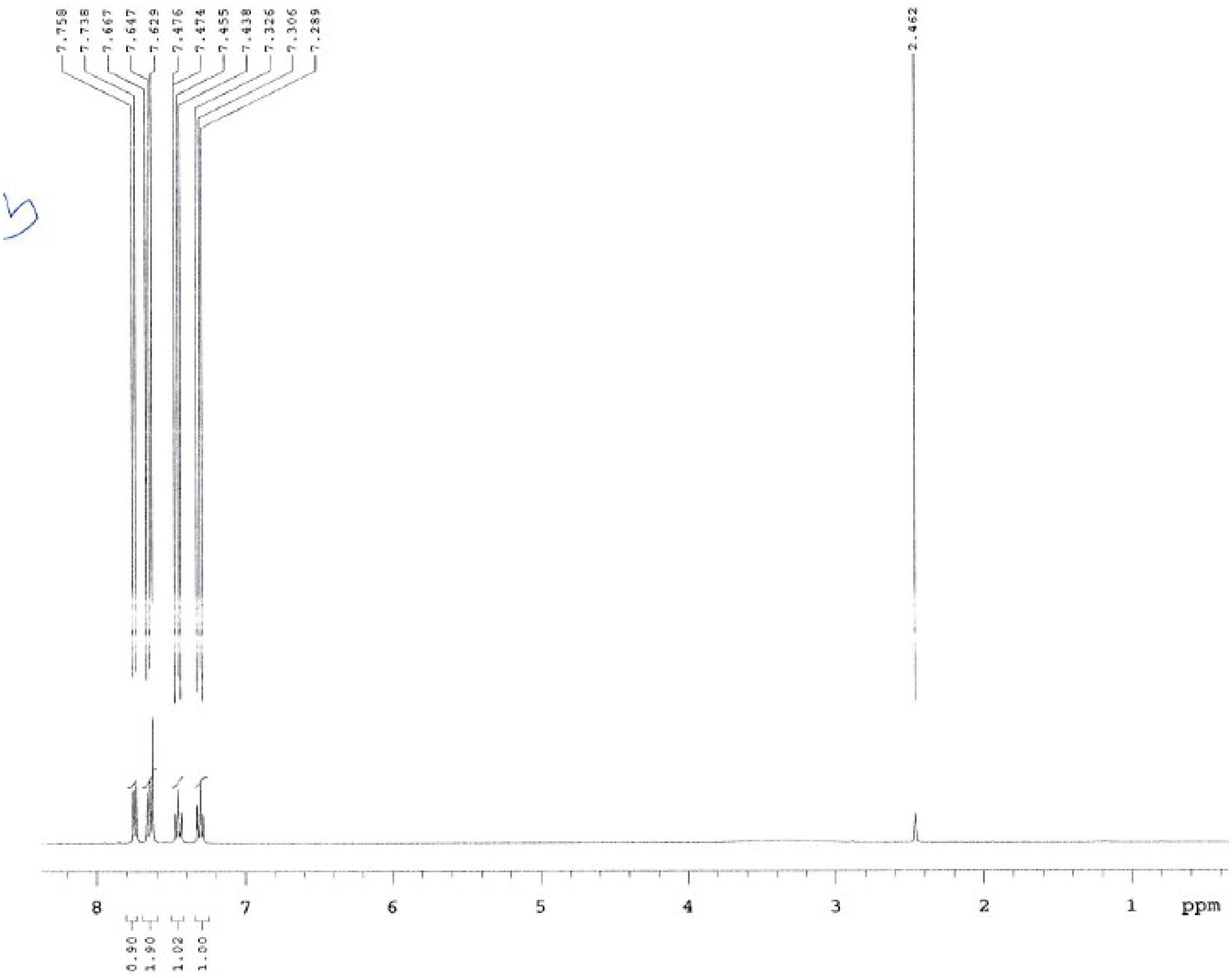

^{1}H NMR spectrum of compound 3j in DMSO-d_6

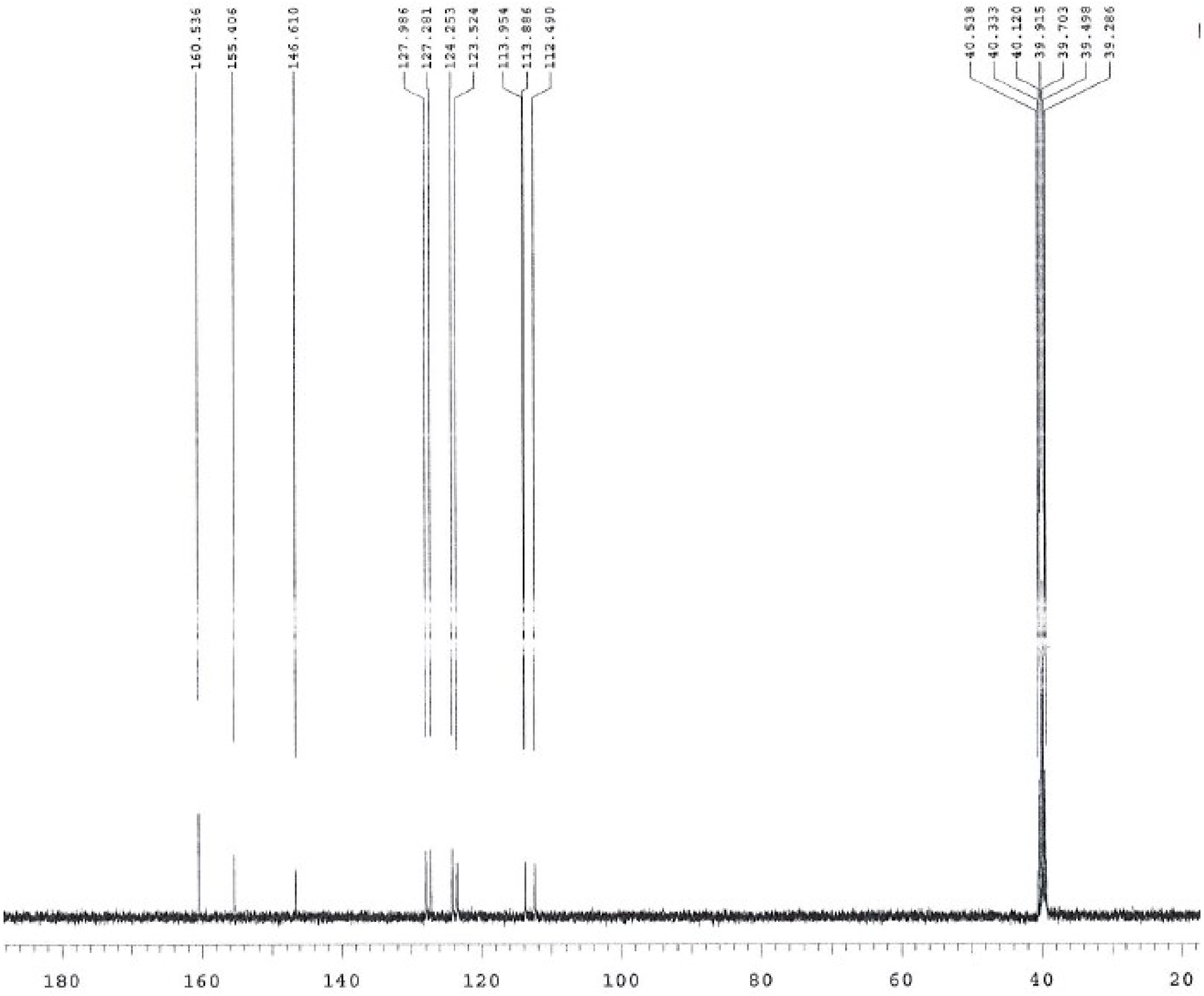

^{13}C NMR spectrum of compound 3j in DMSO-d_6

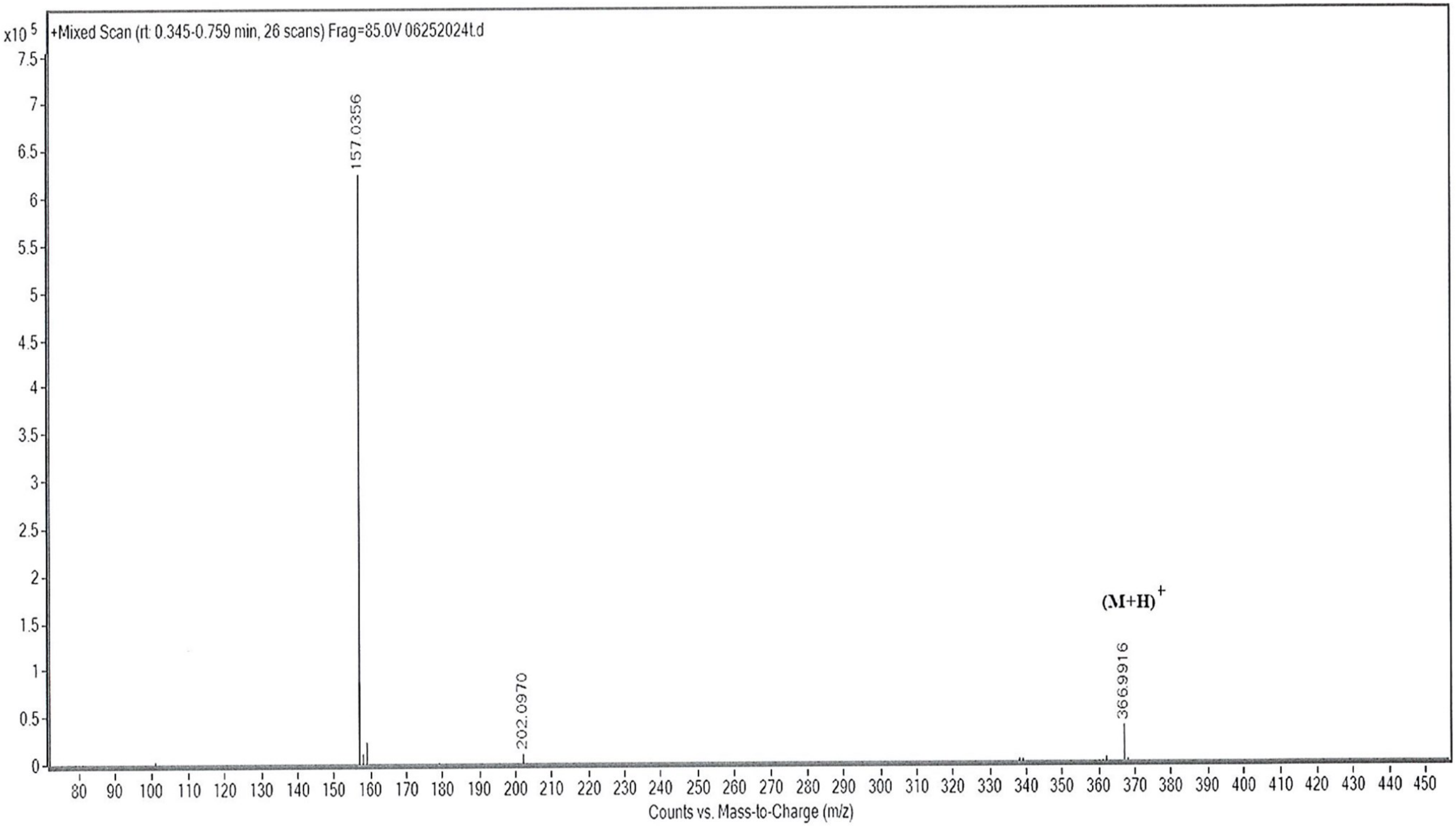

HRMS spectrum of compound 3j

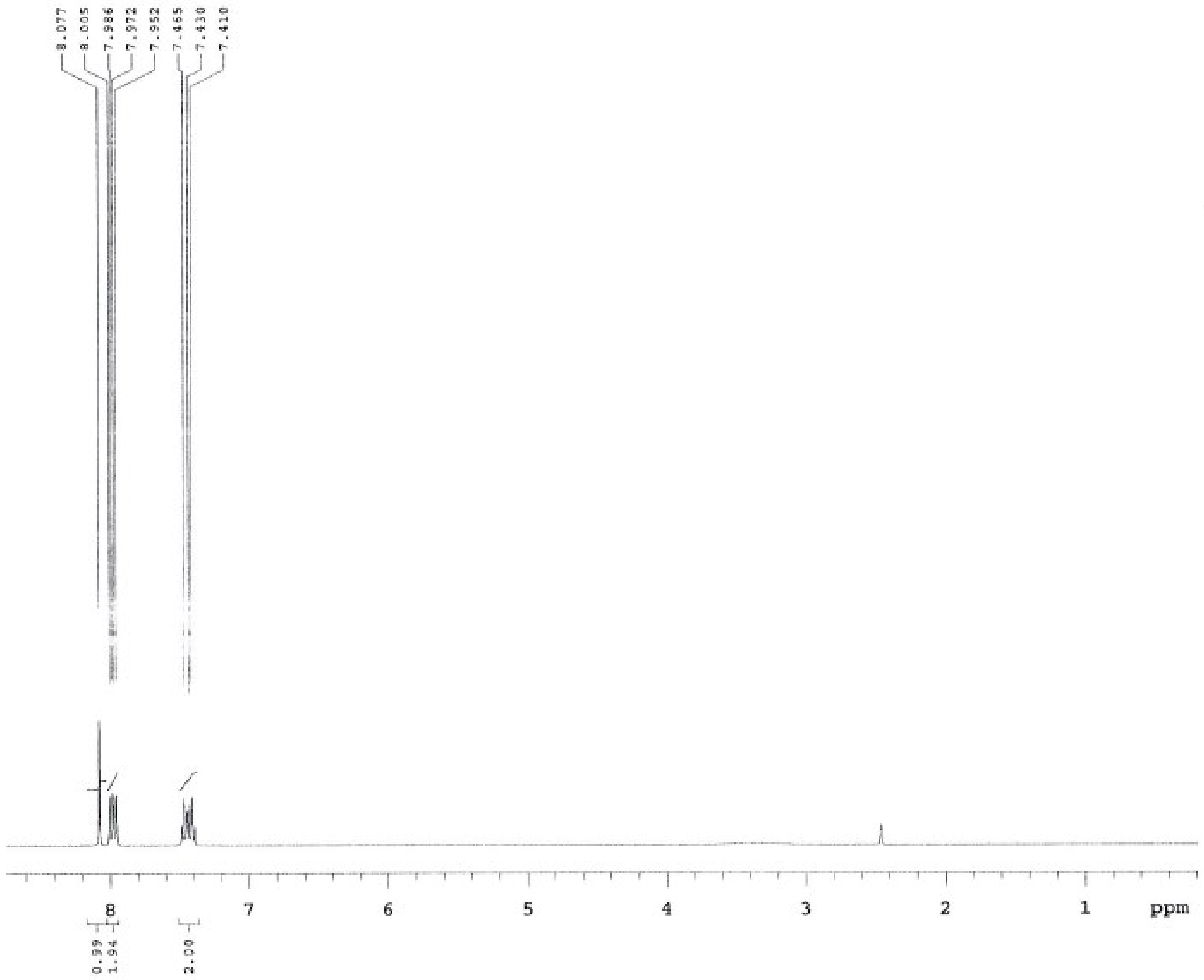

^{1}H NMR spectrum of compound 3k in DMSO-d_6

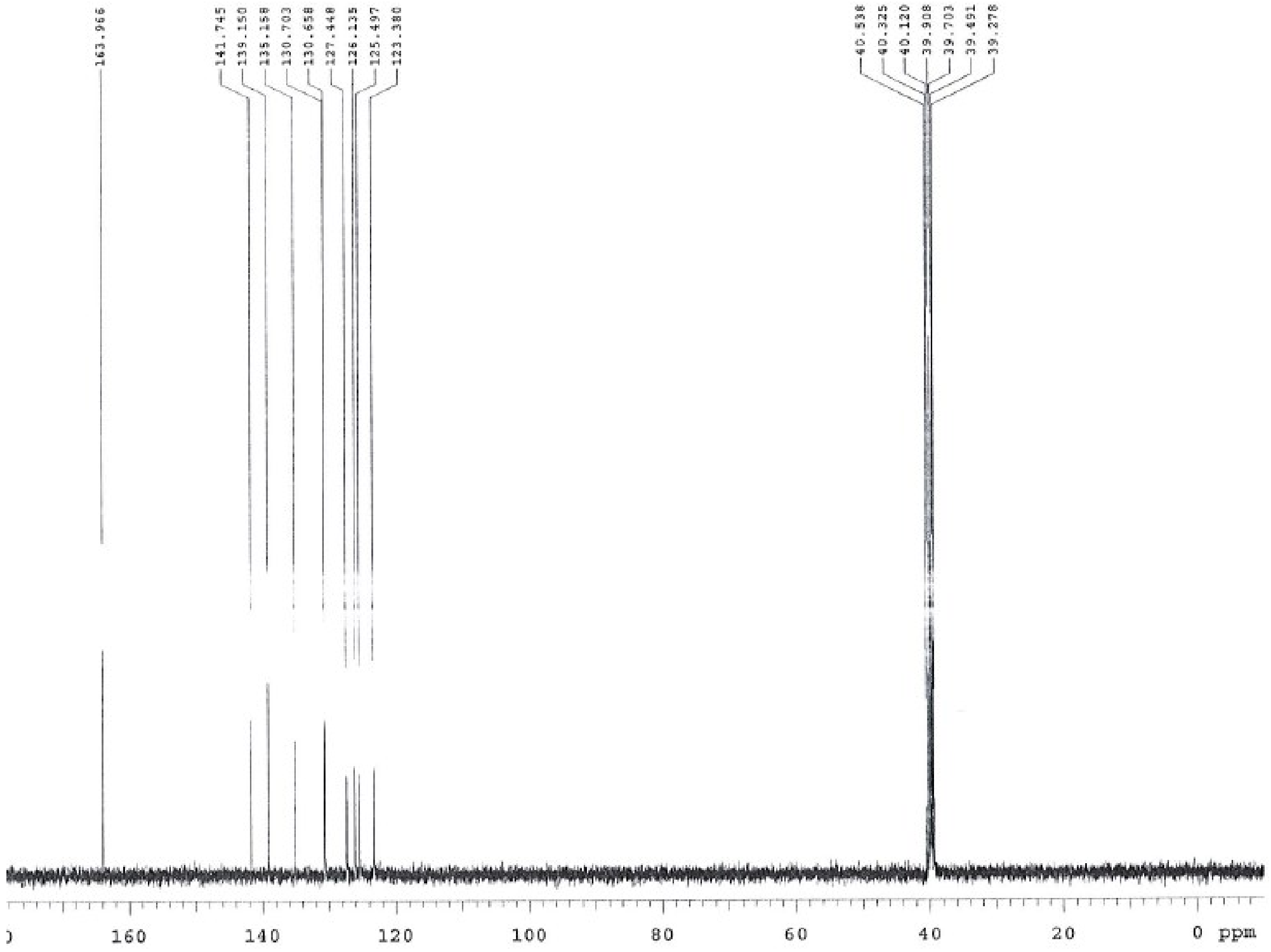

^{13}C NMR spectrum of compound 3k in DMSO-d_6

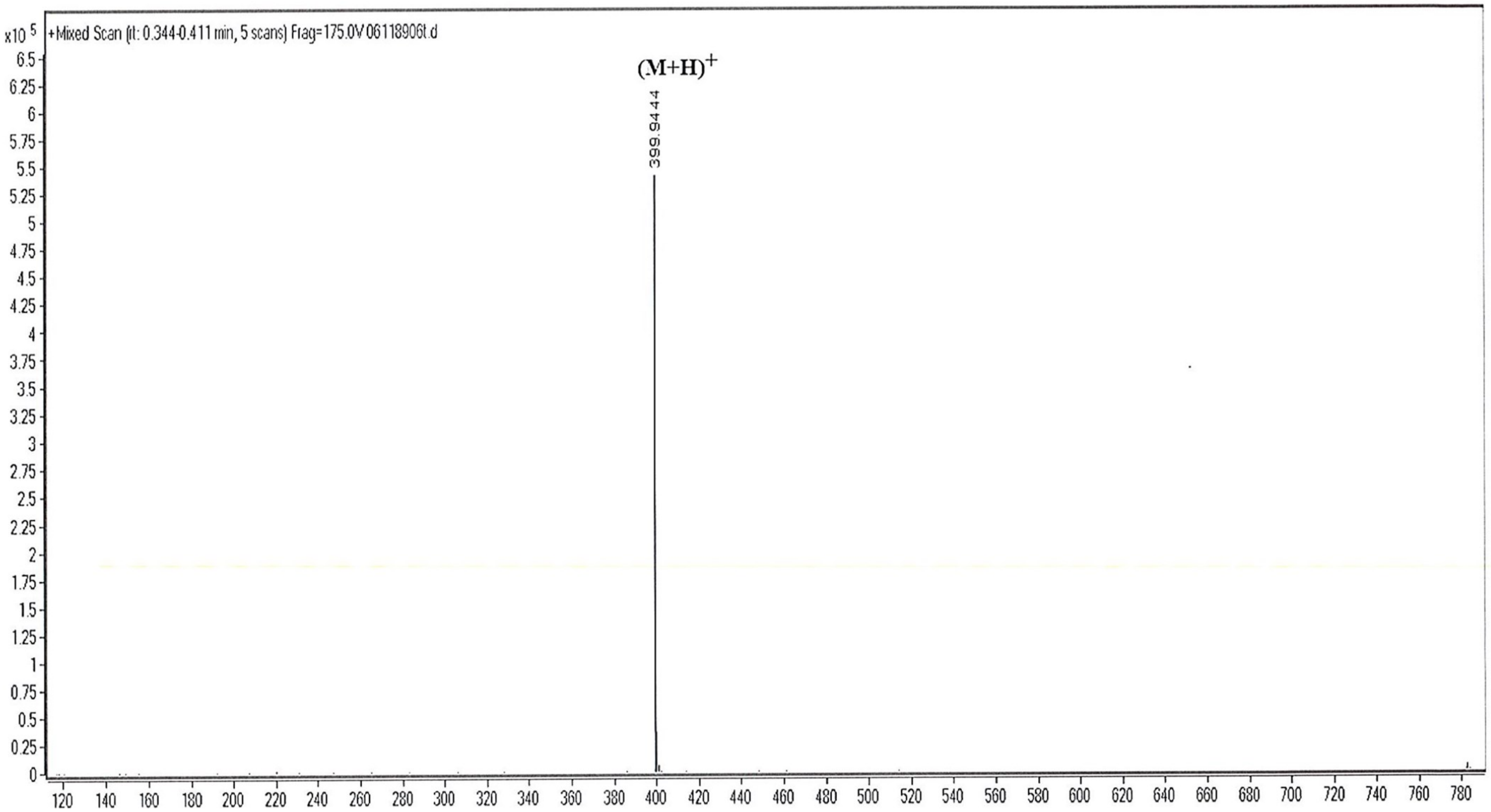

HRMS spectrum of compound 3k

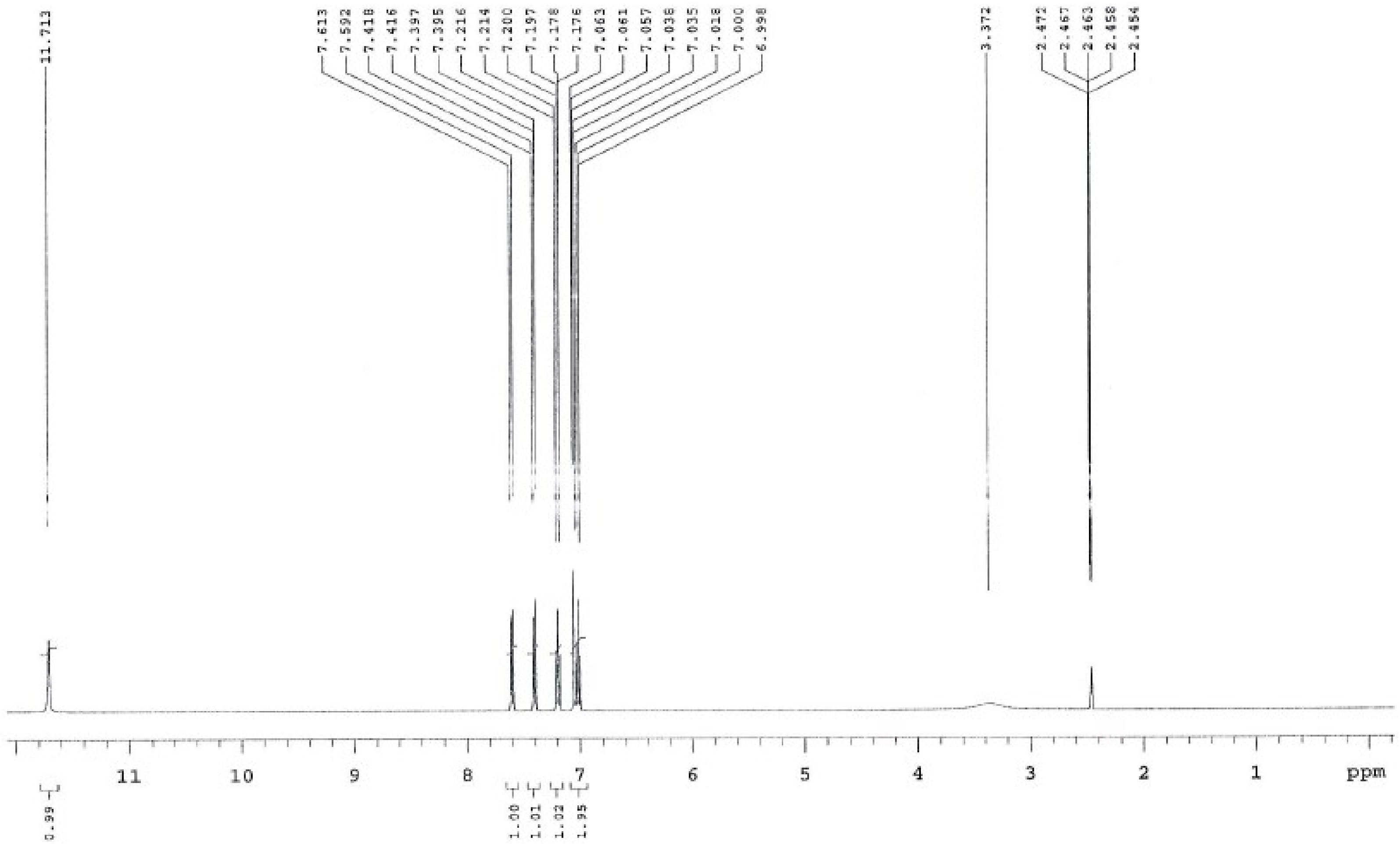

^{1}H NMR spectrum of compound 3l in DMSO-d_6

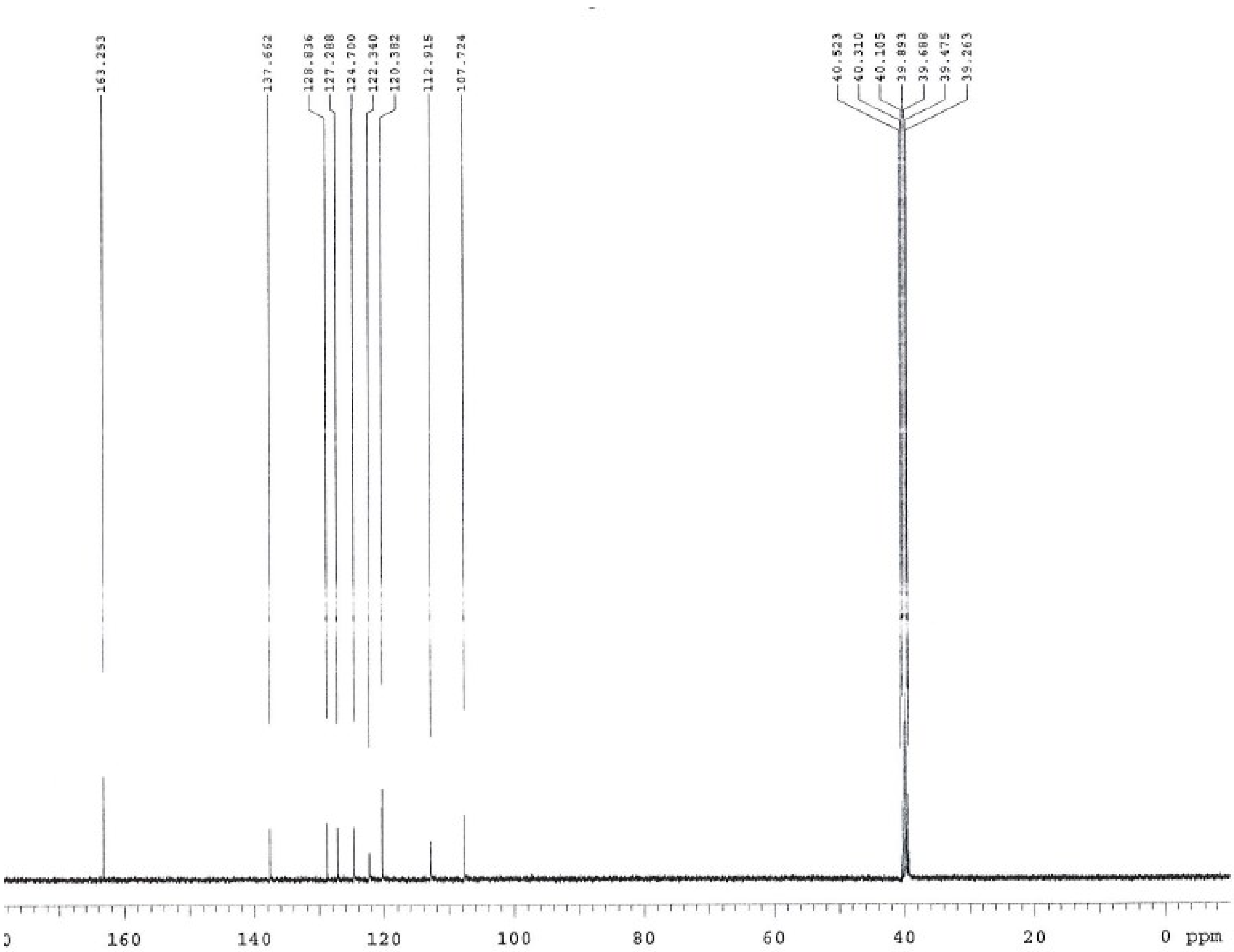

^{13}C NMR spectrum of compound 3l in DMSO-d_6

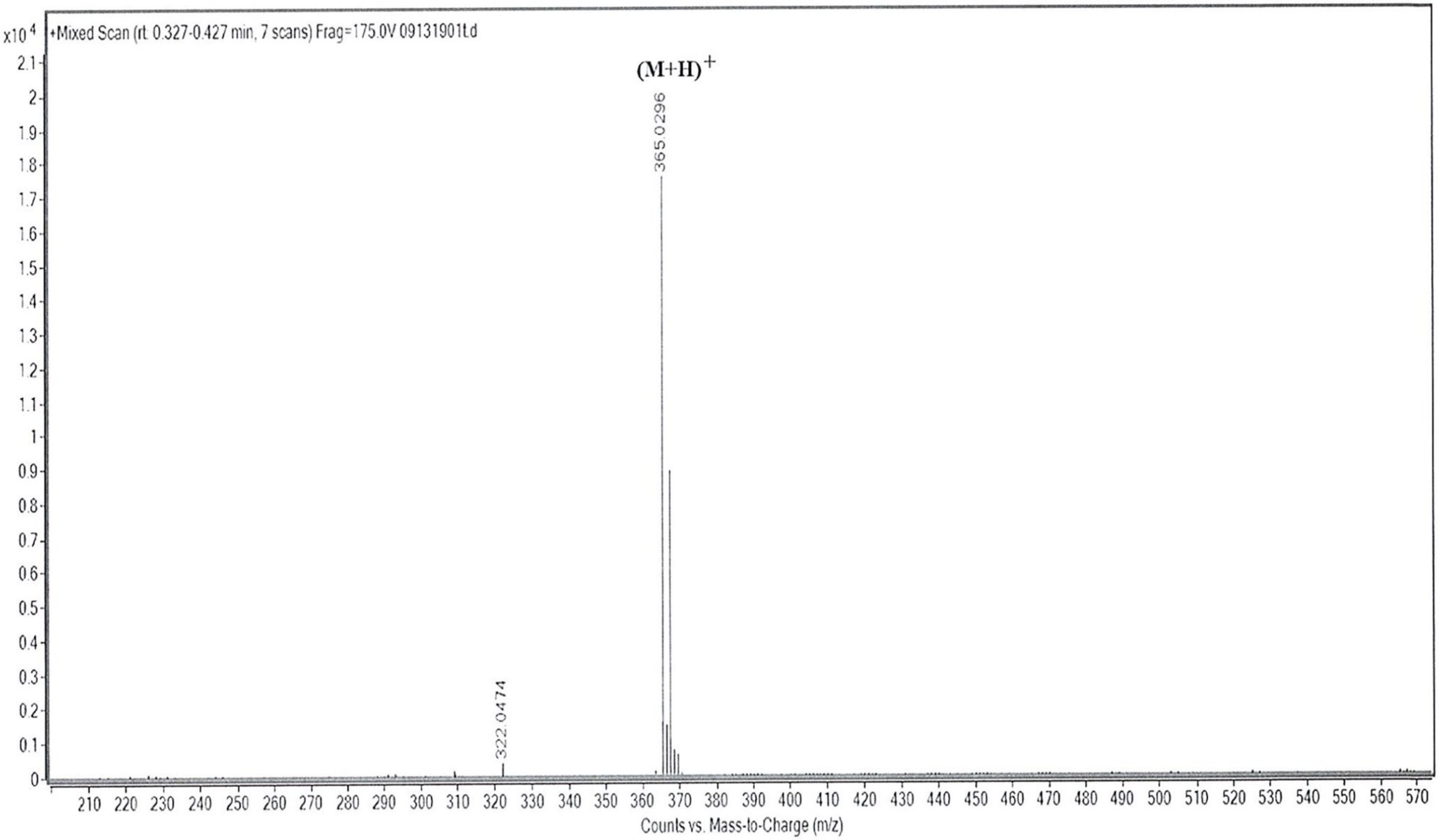

HRMS spectrum of compound 3l

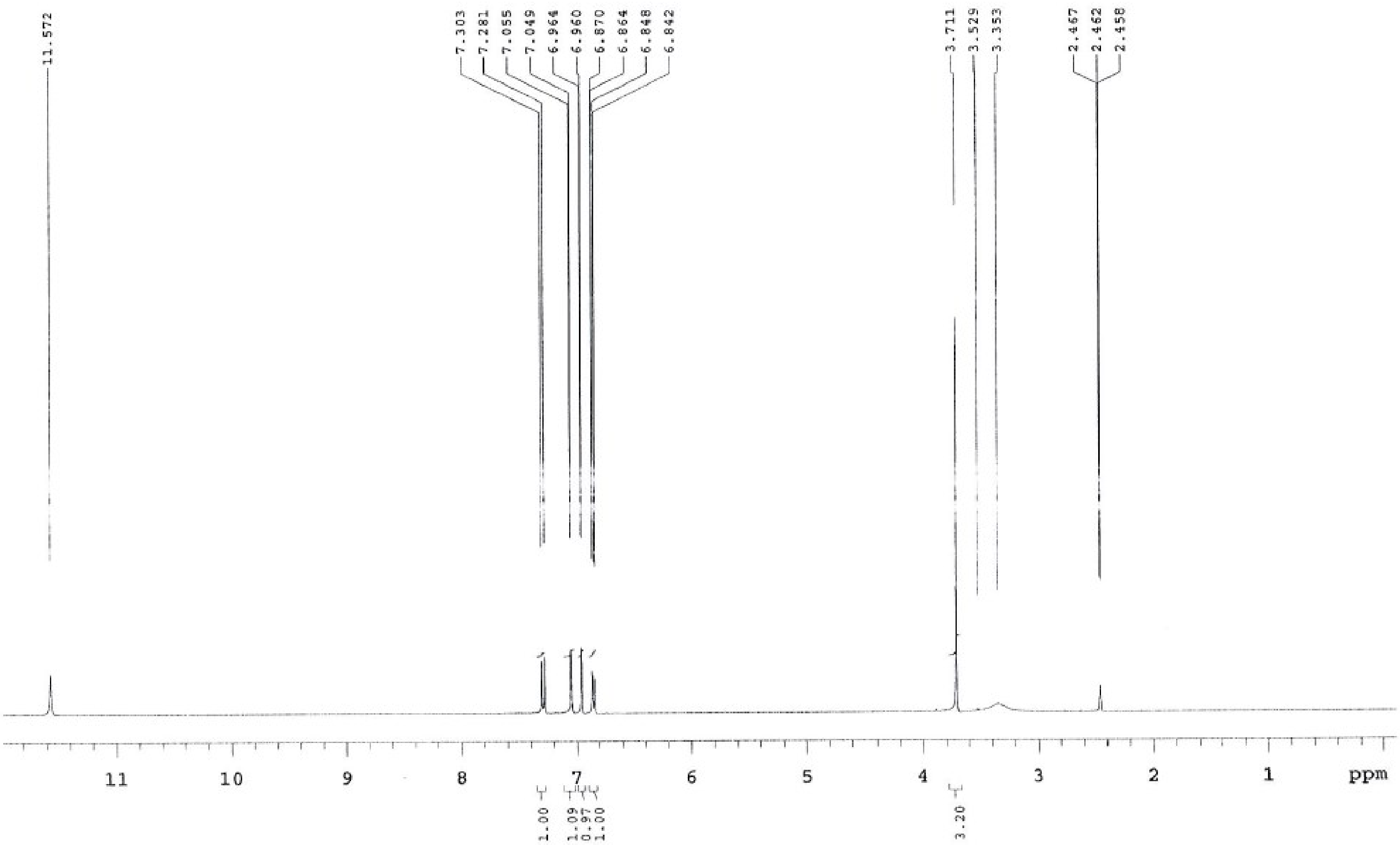

^{1}H NMR spectrum of compound 3m in DMSO-d_6

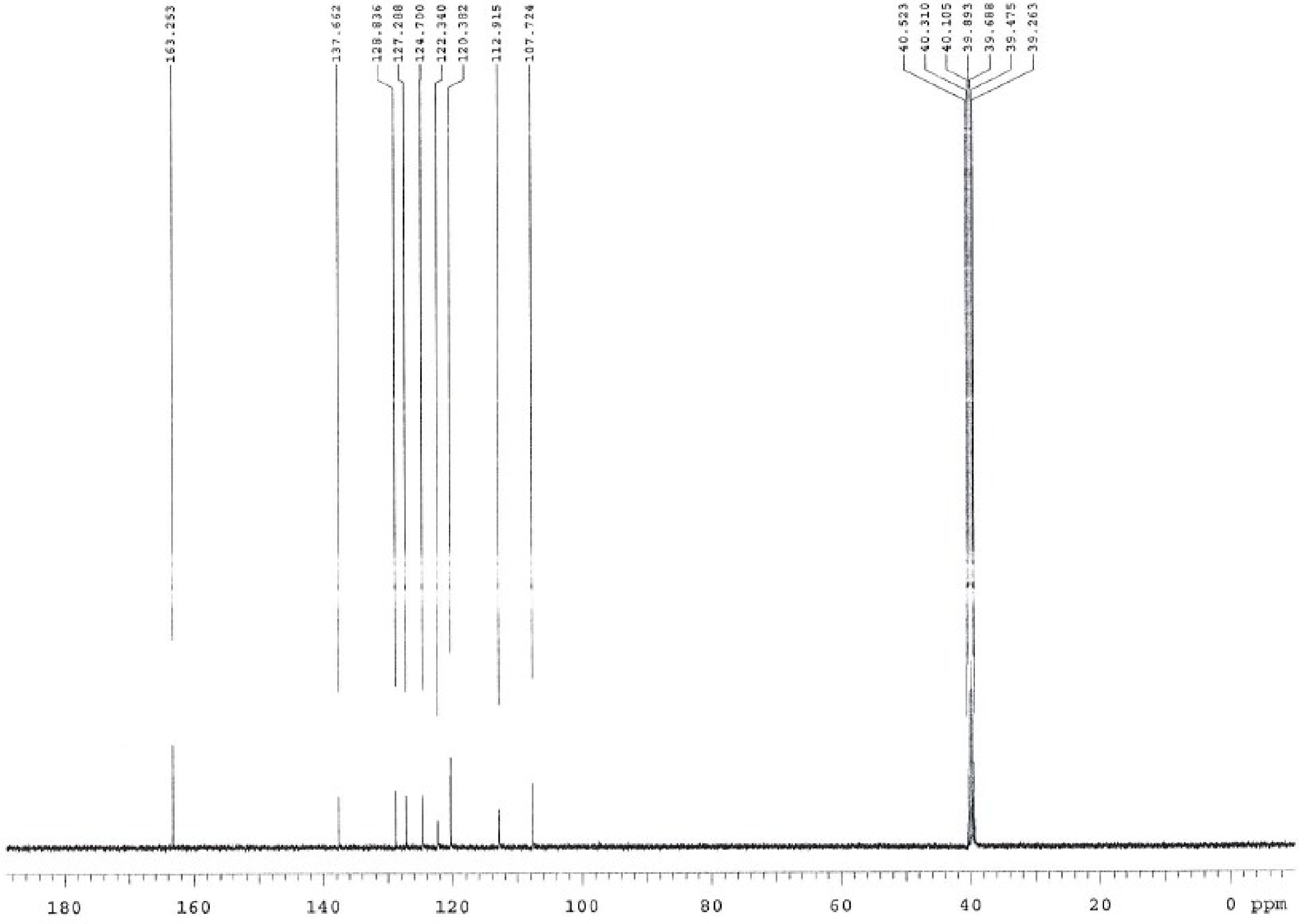

^{13}C NMR spectrum of compound 3m in DMSO-d_6

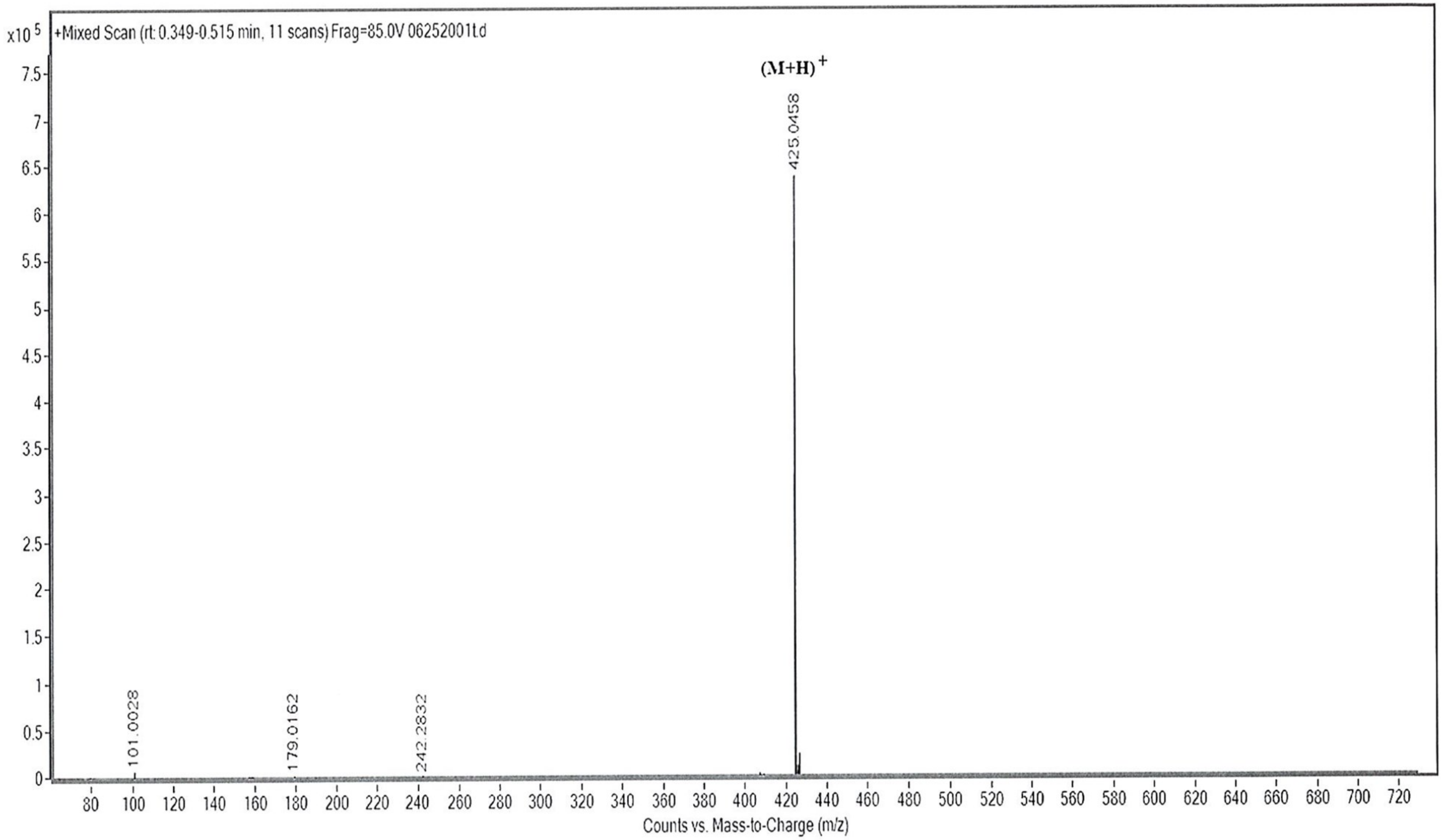

HRMS spectrum of compound 3m

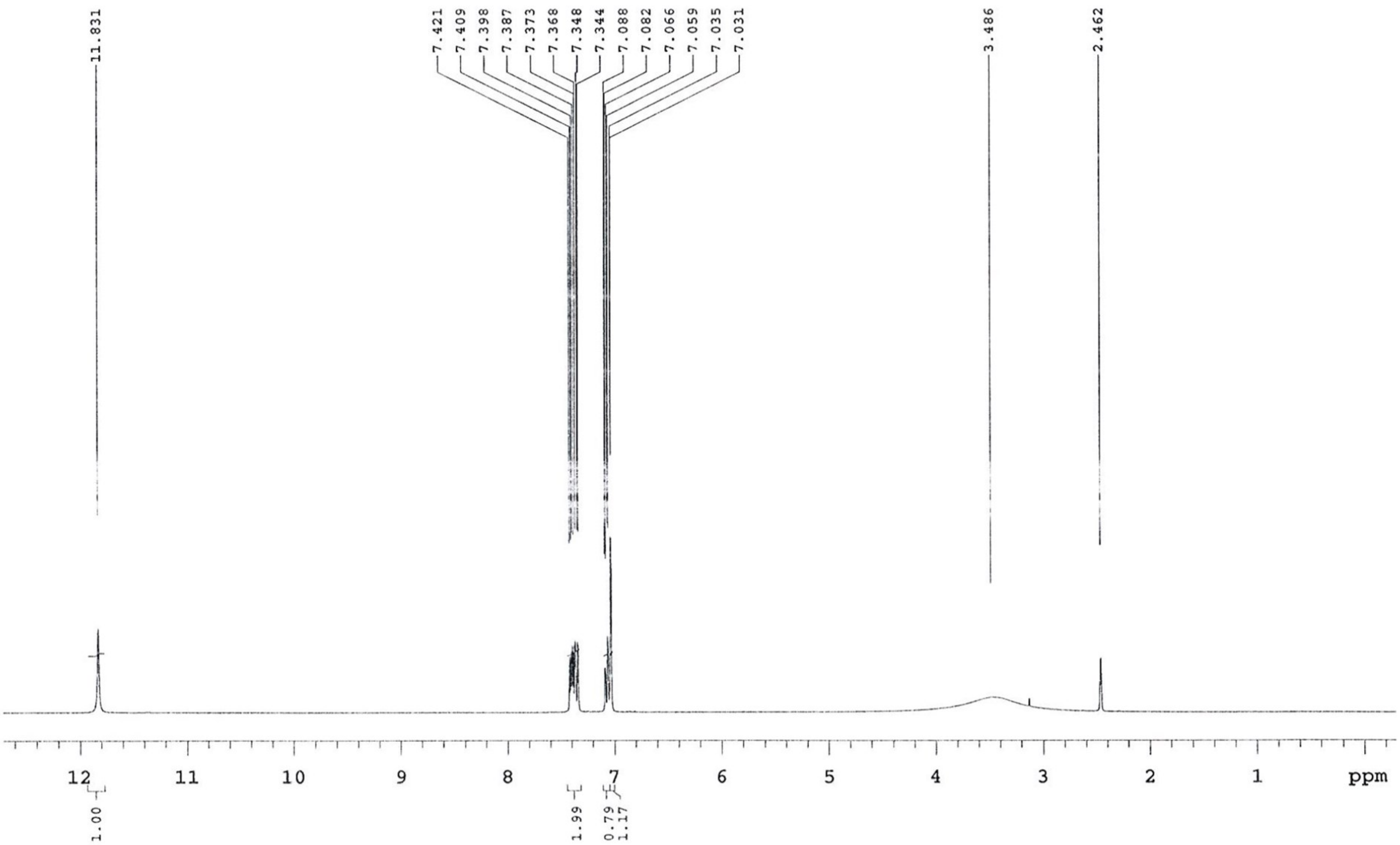

^{1}H NMR spectrum of compound 3n in DMSO-d_6

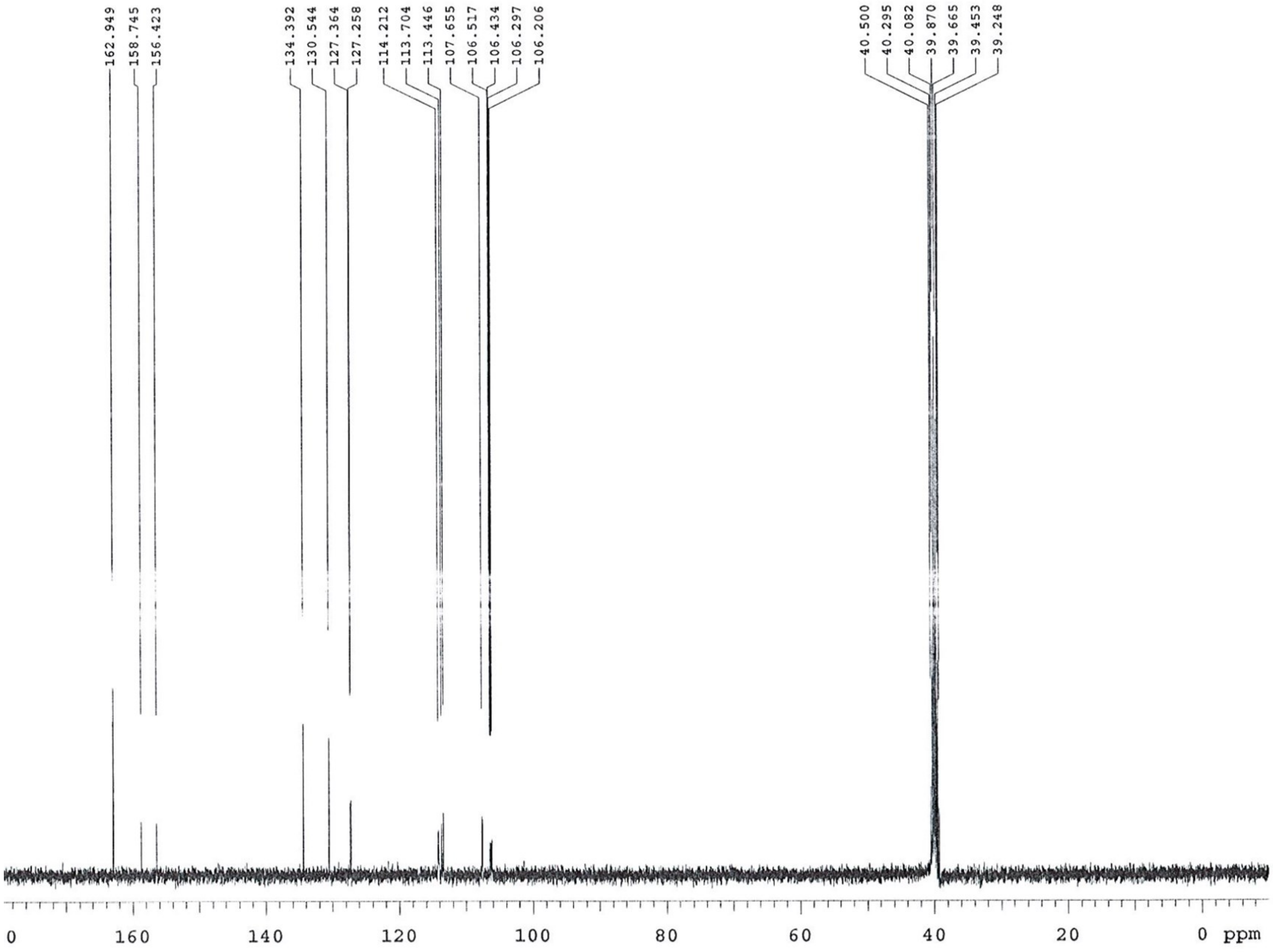

^{13}C NMR spectrum of compound 3n in DMSO-d_6

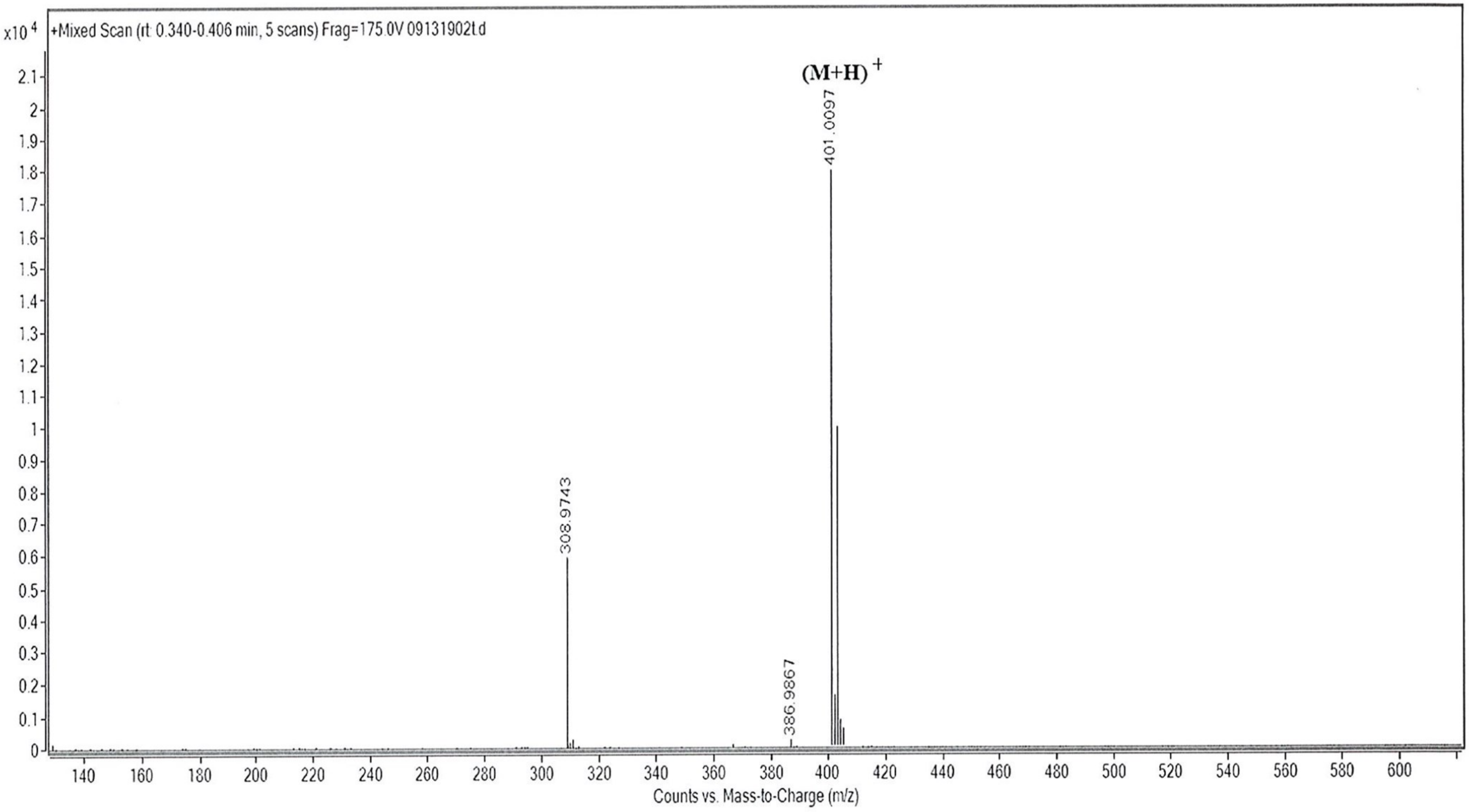

HRMS spectrum of compound 3n
